# Evolution of Intestinal Microbiota of Asphyxiated Neonates Within 1 Week and Its Relationship With Neural Development at 6 Months

**DOI:** 10.3389/fped.2021.690339

**Published:** 2021-08-23

**Authors:** Xiaojiao Zhang, Lili Liu, Wei Bai, Ying Han, Xinlin Hou

**Affiliations:** Department of Pediatrics, Peking University First Hospital, Beijing, China

**Keywords:** intestinal microbiota, evolution, asphyxia, neonates, neural development

## Abstract

**Introduction:** Asphyxia is an emergent condition in neonates that may influence the function of the nervous system. Research has shown that intestinal microbiota is very important for neurodevelopment. Studies regarding the association between gut microbiota and neurodevelopment outcome in asphyxiated newborns remain scarce.

**Objective:** To study the microbial characteristics of asphyxiated neonates within 1 week of life and to investigate their relationship with neural development at 6 months.

**Methods:** The feces produced on days 1, 3, and 5, and the clinical data of full-term neonates with asphyxia and without asphyxia, delivered from March 2019 to October 2020 at Peking University First Hospital, were collected. We used 16S ribosomal deoxyribonucleic acid amplicon sequencing to detect the intestinal microbiota of asphyxiated neonates and neonates in the control group. We followed up asphyxiated neonates for 6 months and used the Ages and Stages Questionnaires-3 (ASQ-3) to evaluate their development.

**Results:** A total of 45 neonates were enrolled in the study group and 32 were enrolled in the control group. On day 1, the diversity and richness of the microflora of the study group were more than those of the control group. Non-metric multidimensional scaling analysis showed significant differences in the microbiota of the two groups on days 1, 3, and 5. At the phylum level, the main microflora of the two groups were not different. At the genus level, the study group had increased relative abundance of *Clostridium_sensu_stricto_1, Lachnoclostridium, Fusicatenibacter*, etc. on day 1. On day 3, the relative abundance of *Clostridium_sensu_stricto_1, Fusicatenibacter*, etc. was still greater than that of the control group, and the relative abundance of *Staphylococcus* was less than that of the control group. On day 5, the relative abundance of *Clostridium_sensu_stricto_1* and *Lachnoclostridium* was still higher than that of the control group, and the relative abundance of *Dubosiella* in the study group was significantly increased. At the species level, on day 3, the relative abundance of *Staphylococcus caprae* in the study group was less than that in the control group. Linear discriminant analysis effect size showed that the microbiota of the study group mainly consisted of *Lachnospiraceae* and *Clostridia* on day 1 and *Clostridia* on day 3. In the control group, *Staphylococcus* was the dominant bacterium on day 3. Neonates in the study group were followed up for 6 months, and the communication score of ASQ-3 was negatively correlated with the relative abundance of *Lachnospiraceae* and *Clostridia* on day 1.

**Conclusion:** The diversity and richness of the microbiota of asphyxiated neonates on the first day of life were significantly increased and mainly consisted of pathogenic flora. *Lachnospiraceae* and *Clostridia* found in neonates with asphyxia on day 1 of life may be related to neural development at 6 months.

## Introduction

Neonatal asphyxia (asphyxia neonatorum) is a pathological state characterized by hypoxemia, hypercapnia, and metabolic acidosis due to intrauterine hypoxia or dyspnea after birth. It occurs due to various antenatal, intrapartum, and postpartum causes (1). With the development of modern medicine, the incidence of asphyxia neonatorum has gradually decreased (2), but there are still some uncontrollable factors that lead to its occurrence. Most asphyxiated neonates recover quickly after effective resuscitation and post-resuscitation maintenance treatment; however, in recent years, studies have found that even if resuscitation was successful and arterial blood gas analysis soon returned to normal, asphyxia neonatorum is still one of the risk factors for the development of autism spectrum disorder (ASD) and attention deficit hyperactivity disorder (3, 4). In recent decades, researchers have realized that microbiota play a very important role in the human body with respect to metabolism, immunity, and the occurrence of diseases related to intestinal flora, such as obesity, irritable bowel syndrome, and ASD (5–7).

With the increasing number of studies, researchers have gradually found that flora is very important in early growth and neural development (8, 9). At present, some researchers have tried to restore the microbiota of neonates delivered by cesarean section by transplanting microorganisms from the mother's feces (10). The brain–gut axis theory suggests that intestinal microflora is largely related to neurodevelopment (11). Gut microbiota can significantly influence cognition and basic behavioral patterns, such as social connection and coping with stress (12). In one study, researchers found that germ-free mice showed more motor activity and less anxiety behavior than normal mice. When the intestinal flora of the germ-free mice was restored, their motor activity and anxiety behavior became similar to those of normal mice (13). In addition, germ-free mice showed impaired social behavior and obvious social avoidance behavior in early life (14), and supplementation of the intestinal microflora of germ-free mice with that of normal mice at the early postnatal stage could restore the abnormal anxious behavior of the germ-free mice, while supplementation at several postnatal weeks could not restore their abnormal anxious behavior (15). Therefore, researchers have hypothesized that intestinal flora may influence early cognition, social behavior, and later adult behavior (12–14). Supplementation with probiotics increases cognition-related fatty acids, such as arachidonic acid and docosahexaenoic acid, among others (16), and researchers have found that probiotics can alleviate symptoms and improve some behavioral abilities in children with ASD, thus providing a new idea for the treatment of ASD (17–20). In addition, probiotic supplementation in adults has been shown to improve mood control and the ability to cope with stress and anxiety (21–23).

Although the exact mechanism by which intestinal flora affects the central nervous system is still unclear, recent studies have found that intestinal bacteria may affect the signal transduction of the nervous system through microbial metabolites, bacteria-like neuropeptides, intestinal endocrine cells, intestinal immune system, and vagus nerve activity, thereby affecting nervous system activity (12, 24). In addition, Diaz Heijtz et al. found that the expression of synaptic plasticity-related genes, such as brain-derived neurotrophic factor, in germ-free mice was significantly lower in the hippocampus and amygdala that are important parts for regulating cognitive and emotion, and the expression of dopamine D1 receptor was increased in the hippocampus (13).

There are many studies on the development and establishment of the microbiota of healthy neonates, but there are only few studies on the characteristics and evolution of the intestinal flora of asphyxiated neonates and their relationship with long-term neurodevelopment. Kigbu et al. found that the intestinal flora of neonates with asphyxia at 3 days after birth was different from that of neonates without asphyxia. Coagulase-negative *Staphylococcus* accounted for a higher proportion of the intestinal flora of neonates without asphyxia (25). However, their study did not investigate the characteristics of intestinal flora and its relationship with neural development in asphyxiated neonates. The purpose of this present study was therefore to explore the characteristics of intestinal microflora of asphyxiated neonates and to understand the relationship between microbial evolution in the first week after birth and neurodevelopmental prognosis at 6 months.

## Materials and Methods

### Study Subjects and Groups

Term neonates diagnosed with asphyxia and term neonates without asphyxia delivered from March 2019 to October 2020 at Peking University First Hospital were recruited for the study.

#### Inclusion Criteria

(1) The study group included neonates who met the diagnostic criteria of neonatal asphyxia (26), while the control group included non-asphyxiated newborns with aspiration pneumonia or elevated non-specific inflammatory indicators during the same period; (2) gestational age was ≥37 and <42 weeks; (3) newborns had no genetic metabolic diseases, severe intracranial hemorrhage, cerebral infarction, digestive tract malformation, etc.; (4) legal guardians agreed for newborns to be enrolled in the study; and (5) legal guardians agreed with the completion of follow-up.

#### Exclusion Criteria

(1) critical clinical conditions; (2) symptoms of infection of the alimentary tract within 1 week, such as necrotizing enterocolitis, hematochezia, and diarrhea; and (3) presence of diseases that may influence neural development during the follow-up period, such as bacterial meningitis, epilepsy, severe brain injury, and genetic metabolic diseases.

#### Ethical Approval

The study was approved by the Ethics Committee of the Peking University First Hospital.

### Methods

#### Clinical Data and Sample Collection

Clinical data, including sex, gestational age, birth weight, Apgar score, pH of umbilical arterial blood, mode of delivery, and feeding, were collected. The neonates in both the study and control groups were all admitted in the hospital while their mothers were not. The mothers of the neonates collected breast milk with a family breast pump, placed it in a sealed plastic bag, froze it at −20°C, and then transported it to the hospital at low temperature, where it was received by specialized personnel. We collected feces of the neonates on days 1, 3, and 5 of life after feeding. The feces were stored in the PSP spin stool DNA plus kit with a stool DNA stabilizer (Tianwei Taida Technology Co., Ltd, Beijing), temporarily stored in a refrigerator at −20°C, and submitted for examination as soon as possible.

#### Intestinal Microbiota Test and Analysis

The V3 hypervariable region of the 16S rRNA gene was amplified by polymerase chain reaction (PCR), sequencing libraries were generated using Illumina TruSeq DNA PCR-Free Library Preparation Kit (Illumina, USA), following the manufacturer's recommendations, and index codes were added. The library quality was assessed on a Qubit@ 2.0 Fluorometer (Thermo Scientific) and Agilent Bioanalyzer 2100 system. Finally, the library was sequenced on an Illumina NovaSeq 6000 platform, and 250-bp paired-end reads were generated. First, we analyzed the alpha diversity of the intestinal flora to estimate flora complexity. The alpha diversity index includes observed_species, Chao1, abundance-based coverage estimator (ACE), Simpson, Shannon, and goods_coverage. The observer_species index was used to evaluate the actual number of operational taxonomic units (OTUs) in the sample. The richness of the intestinal flora was represented by the Chao1 index or ACE index. The Simpson index and Shannon index were both used to estimate the diversity of intestinal microbiota in the sample. The goods_coverage index was used to evaluate the sequencing depth, and the closer its value was to 1, the closer the sequencing depth was to covering all the bacteria in the tested samples. Rarefaction curve, rank abundance, etc. were used to express the alpha diversity visually. Then, we analyzed the beta diversity of microbiota to estimate the microbial community structure. Principal coordinates analysis (PCoA) and non-metric multi-dimensional scaling (NMDS) were used to analyze the differences in microbial community structure among different samples and groups. Finally, we analyzed the microbial differences among different samples and groups at the phylum, genus, and species levels using MetaStat analysis and linear discriminant analysis (LDA) Effect Size (LEfSe).

#### Diagnostic Criteria for Neonatal Asphyxia

For neonates who had umbilical artery blood gas analysis, we used these definitions: mild asphyxia, Apgar score at 1 min ≤7 points or at 5 min ≤7 points with pH of umbilical artery blood <7.2; severe asphyxia, Apgar score at 1 min ≤3 points or at 5 min ≤5 points with umbilical artery blood pH <7.0. For neonates who had no umbilical artery blood gas analysis, we used the following definitions: mild asphyxia, Apgar score ≤7 points; severe asphyxia, Apgar score ≤3 points (26).

#### Follow-Up of Study Group

We followed up the asphyxiated neonates till 6 months of age and used the Ages and Stages Questionnaires-3 (ASQ-3) to evaluate their development. ASQ-3 mainly includes five parts (communication, gross motor, fine motor, problem solving, and personal–social), and each part consists of six specific evaluation contents.

### Statistical Analysis

Statistical Package for the Social Sciences (version 23.0) was used to analyze the data. Measurement data that had normal distribution were expressed as mean ± standard deviation, and *t*-test was used for comparison. Median values were used to represent measurement data that did not have normal distribution, and non-parametric rank sum test was used for comparison. Enumeration data were expressed as percentages, and comparisons between groups were performed using the χ^2^-test. Pearson correlation analysis was used for data with normal distribution; otherwise, Spearman correlation analysis was used. The rank sum test for multiple associated samples was used for the analysis of multiple associated samples. Statistical significance was set at *p* < 0.05.

## Results

### Clinical Features of Newborns

A total of 45 neonates were enrolled in the study group, with 28 males (62.2%) and 25 neonates (55.6%) delivered by cesarean section. Nineteen newborns (42.2%) were mixed-fed (breast milk +formula) and 26 newborns (57.8%) were formula-fed within 1 week after birth. We collected 45 feces samples on day 1, 45 on day 3, and 31 on day 5 (14 neonates were excluded: three cases of diarrhea, four cases of bloody stools, three cases of abdominal distension and fasting, and four cases treated with advanced antibiotics). A total of 32 neonates were enrolled in the control group, with 17 males (53.1%) and 9 neonates (28.1%) delivered by cesarean section. In the control group, 17 newborns (53.1%) were mixed-fed and 15 newborns (46.9%) were formula-fed within 1 week after birth. A total of 32 fecal samples were collected on day 1, 32 on day 3, and 24 on day 5 (after excluding eight neonates: four cases of diarrhea, two cases of bloody stools, and two cases of abdominal distension and fasting). The proportion of breast milk taken by mixed-fed children was 70–80%.

Asphyxia is a risk factor for neonatal infections (27). Neonates in the control group had inhalation pneumonia or increased non-specific inflammatory indicators. The proportion of ampicillin used was the same in the two groups. The proportion of cesarean sections in the study group was significantly higher than that in the control group. There were no significant differences in gestational age, weight, feeding type, or antibiotic use between the groups. Other details are presented in Table 1.

**Table 1:** Clinical features of neonates.

**Clinical features of newborns**	**Study group**	**Control group**	**Statistic**	***p***
Male	28 (62.2%)	17 (53.1%)	0.64	0.43
Gestational age (weeks)	39.5 ± 1.1	39.4 ± 1.1	0.64	0.53
BW (g)	3385.9 ± 373.8	3373.4 ± 320.6	0.15	0.88
1-min Apgar score, median (range)	6 (5–7)	10 (8–10)	−7.81	0.00
5-min Apgar score, median (range)	9 (8–10)	10 (9–10)	−7.06	0.00
Cesarean	25 (55.6%)	9 (28.1%)	5.71	0.017
Formula + breast milk	19 (42.2%)	17 (53.1%)	0.89	0.35
Using ampicillin within 5 days after birth	45 (100%)	30 (93.7%)	0.95	0.33

### Intestinal Microbiota Analysis

After assembling reads, 97,074 tags were measured per sample on average, and 88,177 valid data were obtained on average after quality control. The sequence was clustered into OTUs with 97% identity, and a total of 11,621 OTUs were obtained.

#### Sequencing Depth Analysis

After the OTUs were obtained, a rarefaction curve was drawn to assess whether the current sequencing depth could fully reflect the microbial diversity contained in the samples. The rarefaction curve tended to be flat (as shown in Supplementary Figure A), indicating that the sequencing depth was reasonable. The goods_coverage index of the sample fluctuated at 0.99–1, indicating that the sequencing depth was close to covering all the bacterial communities in the tested samples.

#### Comparison of Alpha Diversity of Fecal Flora in the Study Group at 1, 3, and 5 Days

Comparison of alpha diversity of fecal flora in the study group on different days revealed significant differences in Chao1, ACE, Simpson, and observed_species, indicating that there were differences in microbiota richness and diversity within the study group at 1, 3, and 5 days after birth. Comparison of the alpha diversity index in the study group at 1 and 3 days indicated significant differences in Chao1, Simpson, Observed_species, and ACE indices (*p* < 0.05), suggesting that the diversity and richness of intestinal microbiota in the study group at 1 days were higher than those at 3 days. Comparison of gut flora of the study group at 3 and 5 days postnatally showed no difference in alpha diversity index, as shown in Supplementary Table 1.

#### Comparison of Alpha Diversity Between the Study and Control Groups at 1, 3, and 5 Days

Comparison of alpha diversity between the study and control groups on the first day indicated significant differences in Chao1, Simpson, observed_species, ACE, and Shannon indices (*p* < 0.05). The diversity and richness of microflora in the study group on the first day were higher than those in the control group. There was no significant difference in the alpha diversity index between the study group and the control group at 3 and 5 days. Details of these comparisons are presented in Supplementary Figure B and Supplementary Table 2.

#### Comparison of Beta Diversity Between the Study and Control Groups at 1, 3, and 5 Days

**Figure 1:**
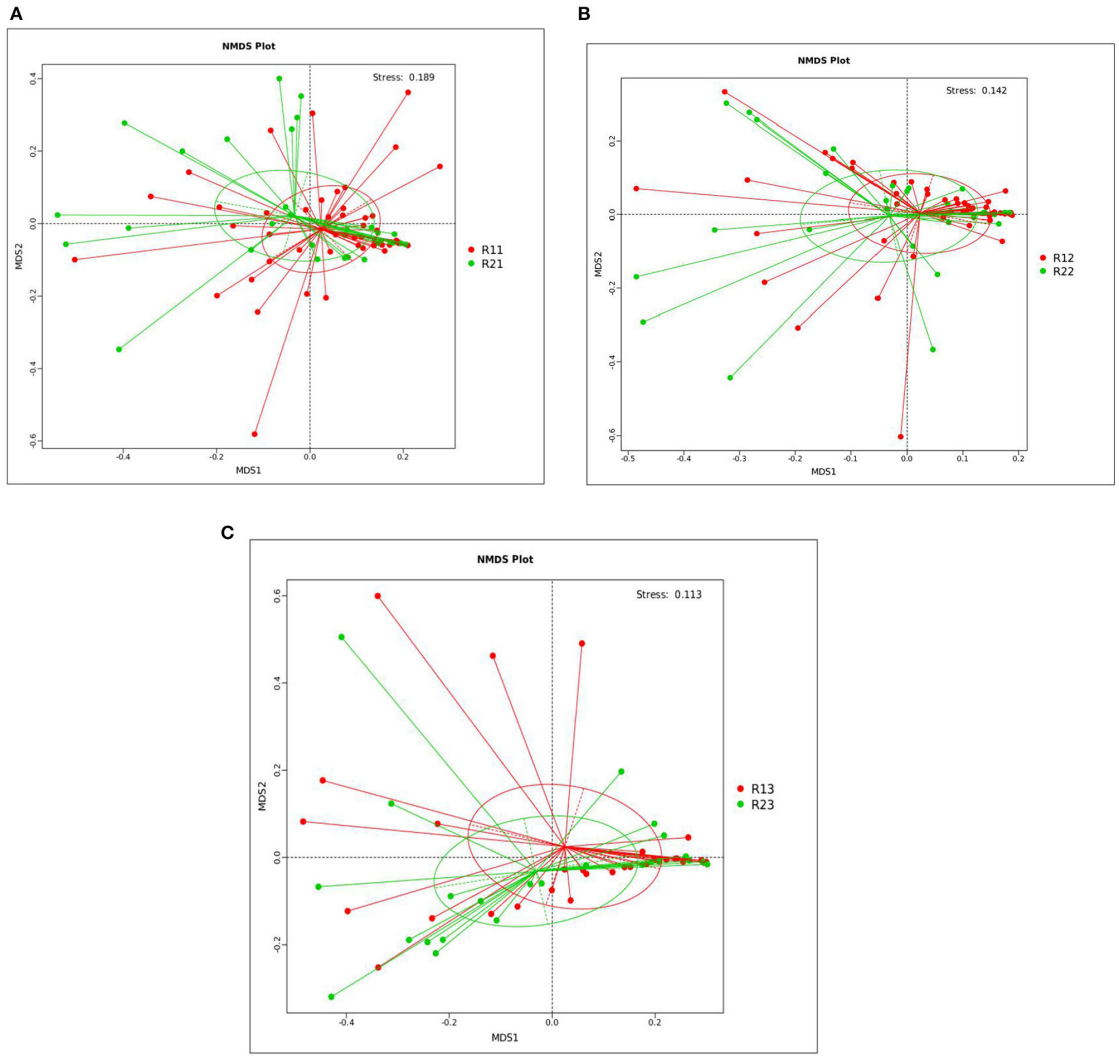
NMDS analysis between the study and control groups on days 1, 3, and 5. **(A)** NMDS analysis between the two groups on day 1. **(B)** NMDS analysis between the two groups on day 3. **(C)** NMDS analysis between the two groups on day 5. Each point in the diagrams represents a sample, the distance between two points indicates the degree of difference, and samples in the same group are represented by the same color. When the stress is <0.2, it indicates that NMDS could accurately reflect the degree of difference between samples. The species information contained in the samples is reflected in the multi-dimensional space in the form of points, while the degree of difference between different samples is reflected in the distance between points, which can reflect the differences between groups and within groups of samples. NMDS, non-metric multi-dimensional scaling; R11, gut flora of the study group on day 1; R21, gut flora of the control group on day 1; R12, gut flora of the study group on day 3; R22, gut flora of the control group on day 3; R13, gut flora of the study group on day 5; R23, gut flora of the control group on day 5.

PCoA showed that the microbiota at 1, 3, and 5 days showed differences between the two groups, but the differences within the two groups were not significant. *R*-value was > 0, and the statistical analysis between groups showed no significant difference, with *p* > 0.05 (Supplementary Figures C1–C6 and Supplementary Table 3). NMDS analysis (see Supplementary Table 4 for the full name) showed that the microbiota of the study group and the control group at 1, 3, and 5 days after birth had significant differences (stress <0.2) (Figure 1).

#### Intestinal Microbiota Analysis

**Figure 2:**
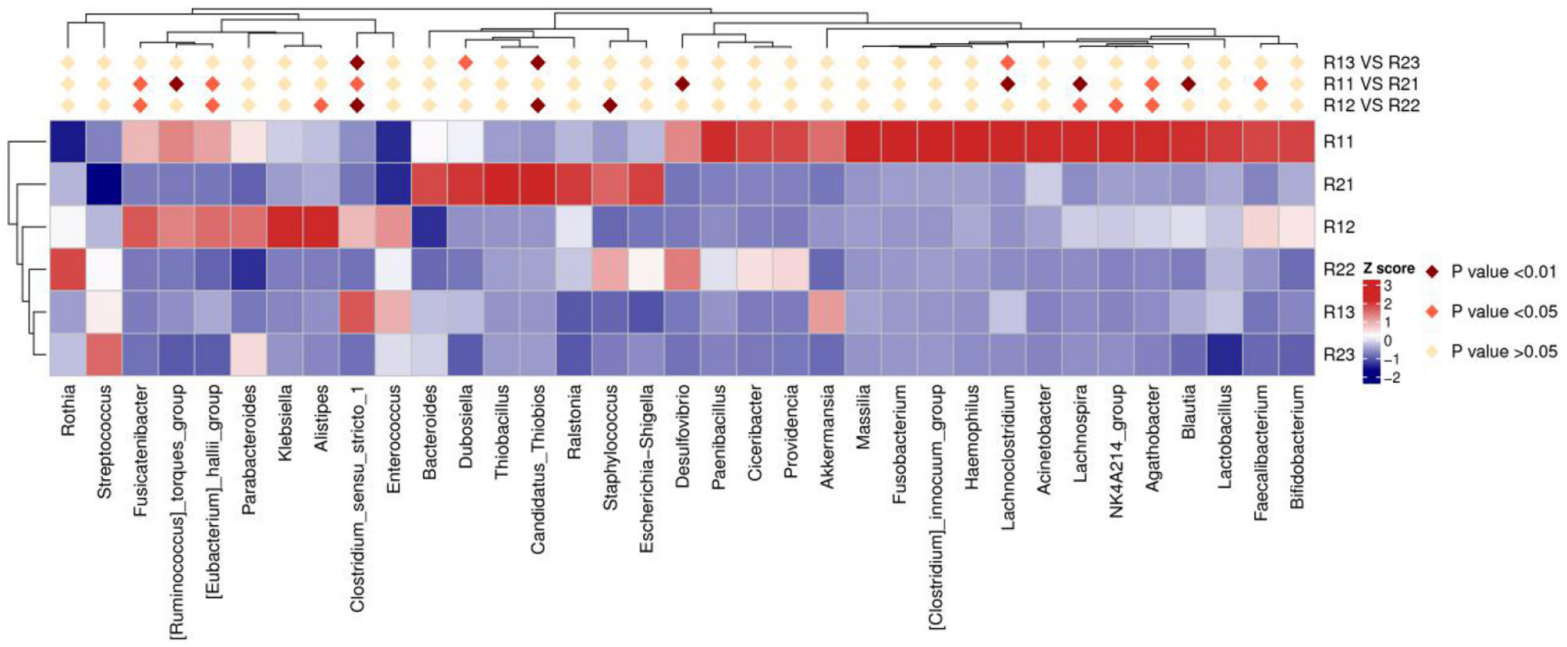
Results of heatmap analysis of species with significant differences at the genus level. R11, gut flora of the study group on day 1; R21, gut flora of the control group on day 1; R12, gut flora of the study group on day 3; R22, gut flora of the control group on day 3; R13, gut flora of the study group on day 5; R23, gut flora of the control group on day 5.

**Figure 3:**
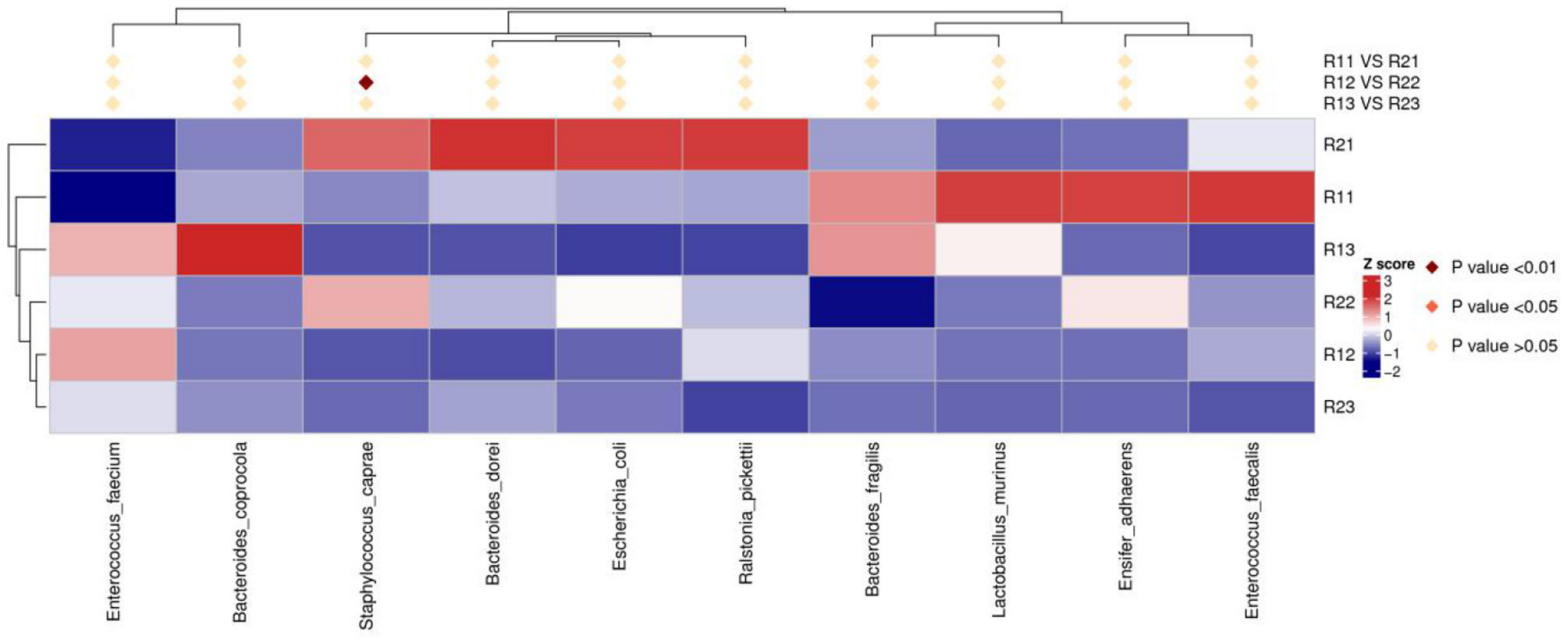
Results of heatmap analysis of species with significant differences at the species level. R11, gut flora of the study group on day 1; R21, gut flora of the control group on day 1; R12, gut flora of the study group on day 3; R22, gut flora of the control group on day 3; R13, gut flora of the study group on day 5; R23, gut flora of the control group on day 5.

At the phylum level, the main microbiota of the two groups were *Firmicutes, Bacteroidetes, Proteobacteria, Actinobacteria, Fusobacteria*, and *Verrucomicrobiota* (Supplementary Figures D1, D2). At the genus level, the main microflora of the two groups were *Enterococcus, Escherichia-Shigella, Streptococcus, Staphylococcus, Bacteroides*, and *Ralstonia*. The study group showed increased relative abundance of *Clostridium_sensu_stricto_1, Lachnoclostridium, Fusicatenibacter, [Ruminococcus]_torques_group, Desulfovibrio, Lachnospira, Agathobacter, Blautia, Faecalibacterium*, and *Eubacterium hallii* groups on day 1 of life. On day 3 of life, the relative abundance of *Clostridium_sensu_stricto_1, Lachnospira, Fusicatenibacter, Agathobacter*, and *[Eubacterium]_hallii_group* was still higher than in the control group, and *Staphylococcus* and *Candidatus_Thiobios* were less than in the control group. At the same time, the *Alistipes NK4A214_group* of the study group was obviously more than that of the control group. On day 5, the relative abundance of *Clostridium_sensu_stricto_1* and *Lachnoclostridium* in the study group was obviously higher than that in the control group, and *Candidatus_Thiobios* was still less than in the control group. In addition, the abundance of *Dubosiella* was higher than that of the control group (Figure 2 and Table 2). At the species level, the main flora of the two groups were *Enterococcus faecium, Enterococcus faecalis, Escherichia coli, Staphylococcus caprae, Ralstonia pickettii, Bacteroides dorei*, and *Bacteroides fragilis*. The study group showed significantly decreased *S. caprae* (2.93 × 10^−4^ vs. 1.23 × 10^−3^; *p* = 0.00) on day 3 of life. On days 3 and 5, there were no significant differences between the two groups (Figure 3).

**Table 2:** Comparison of the relative abundance of microbiota of the study group and control group at the genus level on days 1, 3, and 5.

**Days**	**Taxonomy**	**Study group**	**Control group**	***p***
D1	Clostridium_sensu_stricto_1	3.73 × 10^−4^	5.33 × 10^−5^	0.013
	Lachnoclostridium	1.6 × 10^−4^	2.67 × 10^−5^	0.005
	Fusicatenibacter	5.33 × 10^−5^	2.67 × 10^−5^	0.011
	Desulfovibrio	8 × 10^−5^	1.34 × 10^−5^	0.009
	Lachnospira	5.33 × 10^−5^	0	0.003
	Agathobacter	1.6 × 10^−4^	9.33 × 10^−5^	0.027
	Blautia	2.67 × 10^−4^	8 × 10^−5^	0.008
	Faecalibacterium	3.47 × 10^−4^	2.799 × 10^−4^	0.011
D3	Alistipes	2.67 × 10^−5^	0	0.016
	Lachnospira	5.33 × 10^−5^	0	0.032
	Agathobacter	1.07 × 10^−4^	4 × 10^−5^	0.018
	Staphylococcus	1.87 × 10^−4^	0.0033	0.001
	Clostridium_sensu_stricto_1	1.07 × 10^−4^	2.67 × 10^−5^	0.002
	Fusicatenibacter	5.33 × 10^−5^	0	0.045
D5	Clostridium_sensu_stricto_1	8 × 10^−5^	2.67 × 10^−5^	0.009
	Lachnoclostridium	2.67 × 10^−5^	0	0.02
	Dubosiella	5.33 × 10^−5^	0	0.033

#### LDA Effect Size

**Figure 4:**
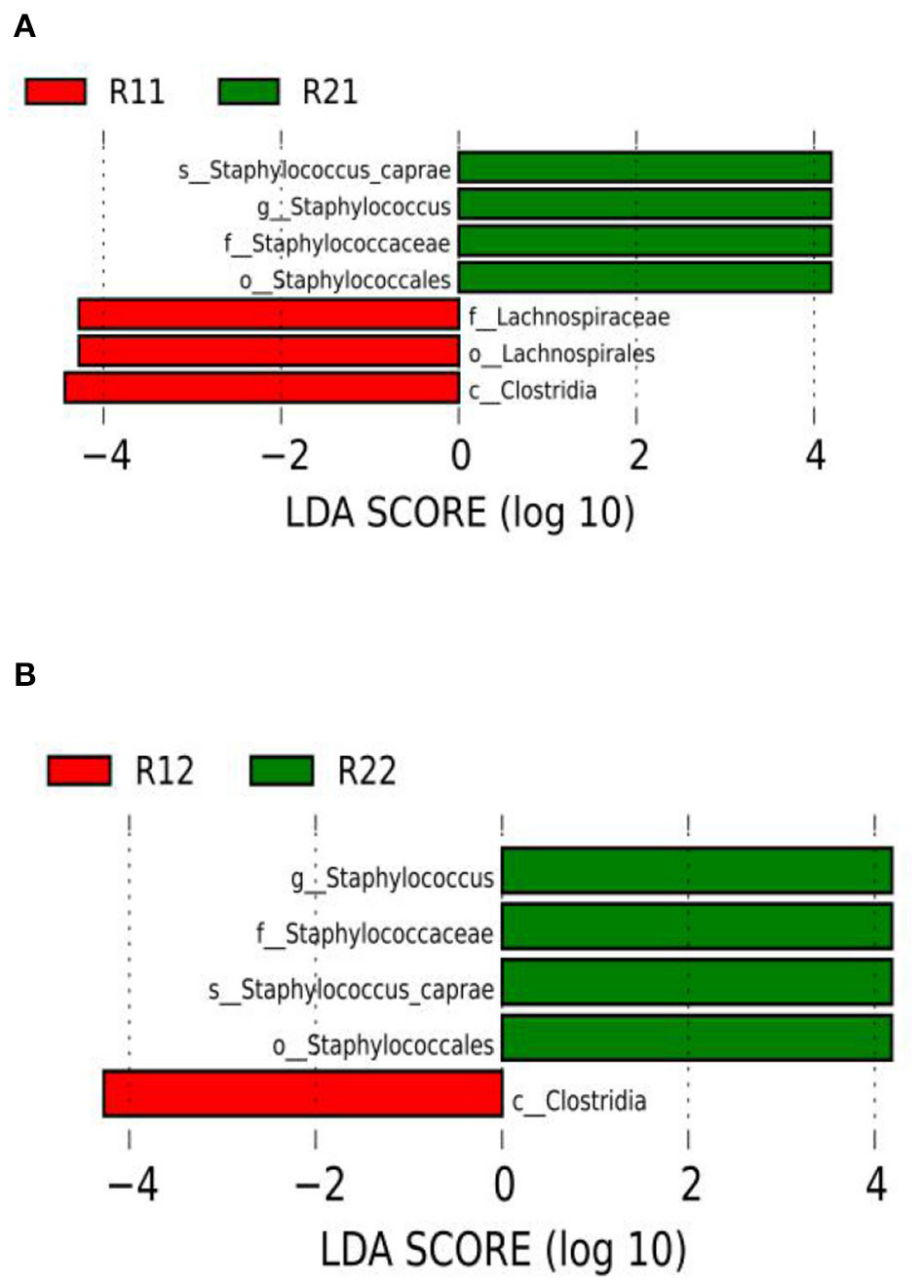
LEfSe comparison between the study and control groups. **(A)** LDA score histogram of differential microbiota of the two groups on day 1 of life. **(B)** LDA score histogram of differential microbiota of the two groups on day 3 of life; c _ represents class level, o_ represents order level, f_ represents family level, g_ represents genus level, and s_ represents species level. The length of the column represents the LDA value, and the greater the value, the greater the influence of the dominant flora. LDA, linear discriminant analysis; LEfSe, linear discriminant analysis effect size; R11, gut flora of the study group on day 1; R21, gut flora of the control group on day 1; R12, gut flora of the study group on day 3; R22, gut flora of the control group on day 3.

We set the LDA score to 3. The dominant microorganisms in the study group on day 1 were *f-lachnospiraceae, o-lachnospirales*, and *c-clostridia*, while on day 3 of life, the dominant microorganisms were *c-clostridia*. The dominant microorganisms in the control group on days 1 and 3 of life were *s-staphylococcus-caprae, g-staphylococcus, f-staphylococcaceae*, and *o-staphylococcales* (Figure 4 and Supplementary Figures E1, E2). There were no dominant microorganisms in either group on day 5.

### Analysis of Intestinal Microbiota in Asphyxiated Neonates With Different Feeding Patterns

In the study group, 45 neonates with asphyxia were enrolled, including 19 (42.2%) who were fed with breast milk and formula after birth, and 26 (57.8%) who were fed with formula only.

#### Comparison of Alpha Diversity in Mixed-Fed Asphyxiated Neonates at 1, 3, and 5 Days After Birth

There were no significant differences in Chao1, ACE, Simpson, observed_species, and Shannon indices at 1, 3, and 5 days, indicating no difference in microbial richness and diversity in mixed-fed neonates at 1, 3, and 5 days (Supplementary Table 5).

#### Comparison of Alpha Diversity in Formula-Fed Asphyxiated Neonates at 1, 3, and 5 Days After Birth

There were statistical differences in observed_species and Shannon indices at 1, 3, and 5 days, indicating differences in flora diversity at 1, 3, and 5 days. Comparison of the alpha diversity index at 1 day and 3 days indicated that there were statistical differences in Shannon and observed_species indices (*p* < 0.05), suggesting that the diversity of intestinal microbiota at 1 day was higher than that at 3 days. Comparison of gut flora at 3 days and 5 days postnatally showed no difference in alpha diversity index (Supplementary Table 6).

#### Comparison of Alpha Diversity Between Mixed-Fed and Formula-Fed Asphyxiated Neonates at 1, 3, and 5 Days

Comparison of alpha diversity between neonates based on the two feeding types on the first day indicated statistical differences in Chao1, observed_species, and ACE indices (*p* < 0.05). The diversity of microflora in neonates who had formula feeding was higher on the first day. There was no significant difference in the alpha diversity index between the two feeding groups at 3 days and 5 days (Supplementary Table 7).

#### Comparison of Beta Diversity Between Mixed-Fed and Formula-Fed Asphyxiated Neonates at 1, 3, and 5 Days

The PCoA analysis showed that at 1 day and 3 days, there were significant differences within the two feeding groups, but the differences between the two groups were not significant. *R*-value was <0, and the statistical analysis between groups showed no significant difference (*p* > 0.05). However, at 5 days, the differences between the two feeding groups was significant; *R* > 0 and *p* < 0.05 (Supplementary Figures F1–F3 and Supplementary Table 8). NMDS analysis showed that at 1, 3, and 5 days after birth, there were significant differences between the two feeding groups (stress <0.2) (Supplementary Figures G1–G3).

#### Intestinal Microbiota Analysis

At the phylum level, the main microbiota of the two feeding groups were not different. At the genus level, the formula-fed neonates showed increased relative abundance of *Clostridium_sensu_stricto_1* on days 1, 3, and 5; decreased *Parabacteroides* on day 1; increased *Ralstonia* and *Escherichia-Shigella* on day 3; and decreased *Streptococcus* and increased *Faecalibacterium* on day 5. At the species level, the formula-fed neonates showed increased *R. pickettii* (1.07 × 10^−4^ vs. 7.60 × 10^−4^, *p* = 0.045) and *E. coli* (5.07 × 10^−4^ vs. 1.45 × 10^−3^, *p* = 0.029) at 3 days (Supplementary Figures H1–H3 and Supplementary Table 9).

#### LEfSe Analysis

We set the LDA score to 4. The dominant microorganisms in formula-fed neonates were *f-Burkholderiaceae, g-Ralstonia, s-Ralstonia pickettii, c-Alphaproteobacteria*, and *o-Rhizobiales* on day 1; *p-Proteobacteria, g-Ralstonia, s-Ralstonia pickettii*, and *o-Enterobacterales* on day 3; and *o-Clostridiales, f-Clostridiaceae*, and *g-Clostridium_sensu_stricto_1* on day 5. The dominant microorganisms in mixed-fed neonates were *f-Lactobacillaceae, g-Lactobacillus*, and *o-Burkholderiales* on day 1; and *g-Streptococcus* and *f-Streptococcaceae* on day 5 (Supplementary Figures J1–J6).

#### Correlation Analysis Between the Dominant Flora of The Study Group and Asq-3 Scores at 6 Months Postnatal Age

We followed up the neonates of the study group until 6 months of age: 43 of them completed the follow-up, so the follow-up prevalence was 95.6%. The assessment was performed by a clinician skilled in the ASQ-3 scoring process. The communication score of ASQ-3 was negatively correlated with relative abundance of *Lachnospiraceae* and *Clostridia* on day 1. There was no correlation between *Clostridia* and ASQ-3 scores on day 3 of life (Table 3).

**Table 3:** Correlation analysis between the dominant microflora of the study group and ASQ-3 scores at 6 months.

**Days and flora**	**ASQ-3 scores**	**Correlation index**	***p***
D1 lachnospirales	Communication	−0.366	0.016
	Gross motor	−0.117	0.466
	Fine motor	−0.136	0.384
	Problem solving	−0.171	0.273
	Personal–social	0.027	0.864
D1 clostridia	Communication	−0.030	0.031
	Gross motor	−0.052	0.739
	Fine motor	−0.161	0.302
	Problem solving	−0.120	0.445
	Personal–social	0.094	0.548
D3 clostridia	Communication	0.055	0.727
	Gross motor	−0.196	0.207
	Fine motor	−0.011	0.942
	Problem solving	−0.155	0.321
	Personal–social	−0.102	0.424

## Discussion

This study examined the characteristics of intestinal microflora of asphyxiated neonates, and the relationship between microbial evolution in the first week after birth and neurodevelopmental prognosis at 6 months. We found that the microbial richness and diversity of the study group were significantly increased on day 1 and mainly consisted of pathogenic flora. The relative abundance of microbiota, such as *Clostridium_sensu_stricto_1* and *Lachnoclostridium*, decreased on day 3 of life. On day 5, the microbial richness and diversity of the study group were not significantly different from those of the control group. *Lachnospirales* and *Clostridia* present in asphyxiated neonates on day 1 of life may have some relationship with neural development at 6 months.

The microbial richness and diversity of the two groups were high on day 1, and there was a temporary decrease around the first week, which is similar to the results of Brandt et al. (28) and Brazier et al. (29). The microbial richness and diversity of asphyxiated neonates were significantly increased compared to those of the control group. This may be because asphyxiated neonates were at increased risk of infection, due to more people involved in their resuscitation, exposure to more drugs and instruments, and longer exposure to the hospital environment (25, 30). It may also be related to the disease status of the neonates themselves, as studies have also found that the richness and diversity of intestinal flora increase under certain disease conditions (31, 32). Studies have found that the nervous system may be affected by the stress response system (24, 33). Since asphyxia is a stressful condition that makes the body's hormone and blood sugar levels to change significantly, we believe that asphyxia may increase the richness and diversity of intestinal microbiota.

We found that, at the phylum level, the dominant intestinal microbiota of the two groups were *Firmicutes, Bacteroidetes, Proteobacteria, Actinobacteria, Fusobacteria*, and *Verrucomicrobiota*, which was similar to findings of previous studies (28, 34). *Firmicutes, Bacteroidetes, Proteobacteria, Actinobacteria*, and *Verrucomicrobiota* are also widely present in the gastrointestinal tract of adolescents and adults (35). At the genus level, the main intestinal microflora of neonates in both the study and control groups consisted of aerobes, facultative anaerobes, and anaerobes, such as *Enterococcus, Escherichia-Shigella, Streptococcus, Staphylococcus, Bacteroides*, and *Ralstonia*. This finding is also consistent with previous research results (25, 28, 36).

At the genus level, the study group showed increased relative abundance of *Clostridium_sensu_stricto_1, Lachnoclostridium, Fusicatenibacter, Desulfovibrio, Lachnospira, Agathobacter, Blautia*, and *Faecalibacterium* on day 1. On day 3, *Clostridium_sensu_stricto_1, Lachnospira, Agathobacter*, and *Fusicatenibacter* were still higher than in the control group. At the same time, the number of *Alistipes* significantly increased. On day 5, *Clostridium_sensu_stricto_1* and *Lachnoclostridium* continued to increase, and *Dubosiella* also increased. *Clostridium_sensu_stricto_1, Alistipes, Lachnoclostridium*, and *Desulfovibrio* are opportunistic pathogens that may cause infection, aggravate inflammation, or worsen clinical conditions (25, 37–41). The exact functions of *Blautia, Lachnospira, Fusicatenibacter, Dubosiella*, and *Agathobacter* are not clear, but some researchers think they may have some relationship with some diseases (32, 42–48). *Faecalibacterium* is beneficial to humans (49, 50).

Summarily, on the first day, the relative abundance of pathogenic bacteria was mainly increased in the study group, while the relative abundance of intestinal pathogenic bacteria on the third and fifth days showed a decreasing trend. Based on clinical experience, the clinical symptoms of asphyxiated neonates improve markedly on day 3 of life, which is an important time point for evaluating neurological outcomes (51). In this study, pathogenic bacteria decreased at 3 days after birth, which was consistent with the time of recovery from clinical symptoms in asphyxiated neonates, but its specific mechanism needs to be further studied. In addition, the differences between the two groups showed a decreasing trend, suggesting that the flora of the neonates with asphyxia may gradually recover with increasing age. The relative abundance of *Staphylococcus* in the control group at 3 days was significantly higher than that in the study group. A number of studies have shown that *Staphylococcus* is the main microbiota of healthy newborns in the early postnatal period (25, 28, 36). At the species level, on the third day, *S. caprae* in our study group was significantly less than in the control group. *S. caprae* is a symbiotic bacterium that is present on human skin, and a coagulase-negative *Staphylococcus*. Its relative abundance was high on the third day after birth in newborns without asphyxia, which is similar to the results of Kigbu et al. (25). At the same time, studies have found that *S. caprae* can inhibit the growth of *Staphylococcus aureus* and is beneficial to the control of infection (52).

Many factors can influence the development of neonatal intestinal microbiota, among which the feeding pattern is one of the most important (53). Breast milk has many benefits. Multiple trace elements, immunoglobulins, and oligosaccharides are present in breast milk, and they play an important role in the establishment and growth of neonatal intestinal microflora (54–57). Different feeding patterns, such as strict breastfeeding, mixed feeding, and formula feeding, have been shown to have different influences on the microbial development of neonates (58). Studies have shown that the dominant flora in the intestinal tract of breastfed infants are *Bifidobacteria, Lactobacillus, Staphylococcus, Megasphaera*, and *Actinobacteria*, while the dominant flora of formula-fed infants are *Clostridiales, Proteobacteria*, and *E. coli* (8, 59–61). In this present study, the feeding types in the study group were mixed feeding and strict formula feeding, and there was no difference in microbial diversity and richness of asphyxiated neonates who were fed with formula and breast milk at 1, 3, and 5 days, while the microbial diversity of formula-fed neonates showed a decreasing trend at 1, 3, and 5 days. Some researchers have also found that the microbial diversity of formula-fed infants decreased compared with that of strictly breastfed infants (59). In this study, the microbial diversity of formula-fed infants significantly increased on day 1, and the flora constitutions of formula-fed and mixed-fed neonates were significantly different on day 5. This was different from the results of Madan et al. who found that at 6 weeks postnatally, the microbial constitution of strictly breastfed infants was significantly different from that of mixed-fed or strictly formula-fed infants, while the microbial constitution was similar between mixed-fed infants and strictly formula-fed infants (62). We hypothesized that the difference may be caused by the feeding period being too short or by asphyxia. LEfSe showed that the dominant microbiota of mixed-fed asphyxiated neonates were *Lactobacillus* and *Streptococcus*, and that *Lactobacillus* increased in the intestinal tract of mixed-fed neonates and in the early postnatal period (58, 63). The strictly formula-fed neonates in the study group had different dominant flora, such as *Ralstonia* and *Clostridium_sensu_stricto_1*, which were similar to those reported by other researchers (8, 61). In addition, *Bifidobacteria* were found in the two feeding groups, but it did not increase significantly, which was different from the findings of some researchers (8, 60). The reasons for this may be related to asphyxia, hospital environment, or other unidentified conditions. Therefore, asphyxia and feeding type may have different influences on the microbial development of full-term neonates, but the exact mechanism is not clear.

Microbiota may have begun to colonize the fetal gut before birth (64). Many studies have found that intestinal flora is likely involved in regulating the development of the nervous system (51). Microbiota may also have a certain influence on human mental activities and behaviors (65) and play a very important role in human health (33). In this study, LEfSe showed that the dominant bacteria in the study group were *Lachnospiraceae* and *Clostridia* on day 1, and *Clostridia* were the dominant bacteria on day 3. The dominant bacterium in the control group was *Staphylococcus* on days 1 and 3 of life.

Neonates in the study group were followed up until 6 months, and the communication score of ASQ-3 negatively correlated with the relative abundance of *Lachnospiraceae* and *Clostridia* on day 1 of life. This suggests that *Lachnospiraceae* and *Clostridia* may be related to neural development at 6 months. Our findings were similar to those of a study that found that the microbiota within the first year of life may be related to cognitive development, especially verbal communication (47). In this study, we found that asphyxia may affect intestinal flora, but the relationship between intestinal flora changes and long-term neural development, and its underlying mechanism need to be further studied.

The study firstly studied the characteristics and evolution of intestinal microflora of asphyxiated neonates <1 week of age, and researched the relationship between specific flora and neural development outcome, providing new insight into the early treatment and improvement of prognosis in asphyxiated newborns. The study has the following limitations: (1) Only neonates with mild asphyxia were included; neonates with severe asphyxia were excluded. (2) The neonates in this study were all hospitalized, and the feeding pattern and use of antibiotics had a certain influence on the intestinal flora. (3) Neural development is influenced by many factors, such as family environment, teaching manners, and society, and we only followed up the neonates in the study group up to 6 months, which could not provide the prognosis of neural development. (4) ASQ-3 is a screening scale. Due to the influence of the novel coronavirus epidemic, it was difficult to use diagnostic scales, such as the Bailey scale, to evaluate neurodevelopment.

## Conclusion

The diversity and richness of the microbiota of asphyxiated neonates on the first day were significantly increased and mainly consisted of pathogenic flora. *Lachnospiraceae* and *Clostridia* found in neonates with asphyxia on day 1 may be related to neural development at 6 months, and neonatal asphyxia may have a serious impact on neurodevelopment, but the current clinical treatment methods for asphyxia are limited. With further research on the microflora of asphyxiated neonates, we hope to provide new insight for early treatment of asphyxia.

## Data Availability Statement

The datasets presented in this study can be found in online repositories. The names of the repository/repositories and accession number(s) can be found at: https://www.ncbi.nlm.nih.gov/Traces/study/?acc=PRJNA721111, PRJNA721111.

## Ethics Statement

The studies involving human participants were reviewed and approved by the Ethics Committee at Peking University First Hospital. Written informed consent to participate in this study was provided by the participants' legal guardian/next of kin.

## Author Contributions

All authors listed have made a substantial, direct and intellectual contribution to the work, and approved it for publication.

## Conflict of Interest

The authors declare that the research was conducted in the absence of any commercial or financial relationships that could be construed as a potential conflict of interest.

## Publisher's Note

All claims expressed in this article are solely those of the authors and do not necessarily represent those of their affiliated organizations, or those of the publisher, the editors and the reviewers. Any product that may be evaluated in this article, or claim that may be made by its manufacturer, is not guaranteed or endorsed by the publisher.

## References

[1] Shao X Ye H Qiu X. Practice of Neonatology. 4th ed. Beijing: People's Medical Publishing House (2015). p. 222.

[2] Wu S Peng F Ding T Tan H Wu Q Yu X. Incidence of neonatal asphyxia and contributing factors for the development of severe asphyxia in Hubei Enshi Tujia and Miao Autonomous Prefecture: a multicenter study. Chin J Contemp Pediatr. (2019) 21:6–10. 10.7499/j.issn.1008-8830.2019.01.002 30675856 PMC7390186

[3] Nu Z Zhang Y Xiong L Huang Y Li H. A study on attention deficit hyperactivity disorder (ADHD) among preschool children in Liuzhou city. Chin J Women Children Health. (2019) 10:28–31. 10.19757/j.cnki.issn1674-7763.2019.01.007

[4] Yin D He Z Duan X Wang Q Liao X Dai N. Meta-analysis of risk factors for autism spectrum diseases in Chinese children. Maternal Child Health Care China. (2018) 12:2877–80. 10.7620/zgfybj.j.issn.1001-4411.2018.12.81

[5] Damms-Machado A Mitra S Schollenberger AE Kramer KM Meile T. Effects of surgical and dietary weight loss therapy for obesity on gut microbiota composition and nutrient absorption. Biomed Res Int. (2015) 2015:806248. 10.1155/2015/806248 25710027 PMC4330959

[6] Fan WT Ding C Xu NN Zong S Ma P Gu B. Close association between intestinal microbiota and irritable bowel syndrome. Eur J Clin Microbiol Infect Dis. (2017) 36:2303–17. 10.1007/s10096-017-3060-2 28785822

[7] Li Q Han Y Dy ABC Hagerman RJ. The gut microbiota and autism spectrum disorders. Front Cell Neurosci. (2017) 11:120. 10.3389/fncel.2017.00120 28503135 PMC5408485

[8] Heijtz DR. Fetal, neonatal, and infant microbiome: perturbations and subsequent effects on brain development and behavior. Semin Fetal Neonatal Med. (2016) 21:410–7. 10.1016/j.siny.2016.04.012 27255860

[9] Douglas-Escobar M Elliott E Neu J. Effect of intestinal microbial ecology on the developing brain. JAMA Pediatrics. (2013) 167:374–9. 10.1001/jamapediatrics.2013.497 23400224

[10] Korpela K Helve O Kolho KL Saisto T Skogberg K Dikareva E. Maternal fecal microbiota transplantation in cesarean-born infants rapidly restores normal gut microbial development: a proof-of-concept study. Cell. (2020) 183:324–34.e5. 10.1016/j.cell.2020.08.047 33007265

[11] Bercik P Denou E Collins J Jackson W Lu J Jury J. The intestinal microbiota affect central levels of brain-derived neurotropic factor and behavior in mice. Gastroenterology. (2011) 141:599–609. 10.1053/j.gastro.2011.04.052 21683077

[12] Dinan TG Stilling RM Stanton C Cryan JF. Collective unconscious: how gut microbes shape human behavior. J Psychiatr Res. (2015) 63:1–9. 10.1016/j.jpsychires.2015.02.021 25772005

[13] Heijtz RD Wang S Anuar F Qian Y Bjorkholm B Samuelsson A. Normal gut microbiota modulates brain development and behaviour. Proc Natl. Acad Sci USA. (2011) 108:3047–52. 10.1073/pnas.1010529108 21282636 PMC3041077

[14] Desbonnet L Clarke G Shanahan F Dinan TG Cryan JF. Microbiota is essential for social development in the mouse. Mol Psychiatry. (2013) 19:146–8. 10.1038/mp.2013.65 23689536 PMC3903109

[15] Neufeld K-AM Kang N Bienenstock J Foster JA. Effects of intestinal microbiota on anxiety-like behavior. Commun Integr Biol. (2011) 4:492–4. 10.4161/cib.15702 21966581 PMC3181531

[16] Wall R Marques TM O'Sullivan O Ross RP Shanahan F Quigley EM. Contrasting effects of *Bifidobacterium breve* NCIMB 702258 and *Bifidobacterium breve* DPC 6330 on the composition of murine brain fatty acids and gut microbiota. Am J Clin Nutr. (2012) 95:1278–87. 10.3945/ajcn.111.026435 22492373

[17] Parracho HMRT Gibson GR Knott F Bosscher D Kleerebezem M McCartney AL. A double blind, placebo-controlled, crossover-designed probiotic feeding study in children diagnosed with autistic spectrum disorders. Int J Probiot Prebiot. (2010) 5:69–74.

[18] Kaluzna-Czaplinska J Blaszczyk S. The level of arabinitol in autistic children after probiotic therapy. Nutrition. (2012) 28:124–6. 10.1016/j.nut.2011.08.002 22079796

[19] Santocchi E Guiducci L Fulceri F Billeci L Buzzigoli E Apicella F. Gut to brain interaction in autism spectrum disorders: a randomized controlled trial on the role of probiotics on clinical, biochemical and neurophysiological parameters. BMC Psychiatry. (2016) 16:183. 10.1186/s12888-016-0887-5 27260271 PMC4893248

[20] Kang DW Adams JB Gregory AC Borody T Chittick L Fasano A. Microbiota Transfer Therapy alters gut ecosystem and improves gastrointestinal and autism symptoms: an open-label study. Microbiome. (2017) 5:10. 10.1186/s40168-016-0225-7 28122648 PMC5264285

[21] Wang H Braun C Murphy EF Enck P. *Bifidobacterium longum* 1714™ strain modulates brain activity of healthy volunteers during social stress. Am J Gastroenterol. (2019) 114:1152–62. 10.14309/ajg.0000000000000203 30998517 PMC6615936

[22] Tillisch K Labus J Kilpatrick L Jiang Z Stains J Ebrat B. Consumption of fermented milk product with probiotic modulates brain activity. Gastroenterology. (2013) 144:1394–401. 10.1053/j.gastro.2013.02.043 23474283 PMC3839572

[23] Tran N Zhebrak M Yacoub C Pelletier J Hawley D. The gut-brain relationship: Investigating the effect of multispecies probiotics on anxiety in a randomized placebo-controlled trial of healthy young adults. J Affect Disord. (2019) 252:271–7. 10.1016/j.jad.2019.04.043 30991255

[24] Osadchiy V Martin CR Mayer EA. The gut–brain axis and the microbiome: mechanisms and clinical implications. Clin Gastroenterol Hepatol. (2019) 17:322–32. 10.1016/j.cgh.2018.10.002 30292888 PMC6999848

[25] Kigbu A Orimadegun AE Tongo OO Odaibo GN Olaleye DO Akinyinka OO. Intestinal bacterial intestinal bacterial colonization in the first 2 weeks of life of Nigerian neonates using standard culture methods. Front Pediatrics. (2016) 4:139. 10.3389/fped.2016.00139 28083526 PMC5186768

[26] Group of Neonatal Neonatal Resuscitation Society of Perinatal Medicine Chinese Medical Association. Neonatal asphyxia: consensus recommendations for diagnosis. Chin J Perinat Med. (2016) 19: 3–6. 10.3760/cma.j.issn.1007-9408.2016.01.002

[27] Chen B Sun L. Analysis of risk factors of NICU neonatal infections by Logistic regression model. China Morden Doctor. (2016) 54:44–6.

[28] Brandt K Taddei CR Takagi EH Oliveira FF Duarte RTD Irino I. Establishment of the bacterial fecal community during the first month of life in Brazilian newborns. Clinics. (2012) 67:113–23. 10.6061/clinics/2012(02)05 22358235 PMC3275115

[29] Brazier L Elguero E Koumavor CK Renaud N Prugnolle F Thomas F. Evolution in fecal bacterial/viral composition in infants of two central African countries (Gabon and Republic of the Congo) during their first month of life. PLoS ONE. (2017) 12:e0185569. 10.1371/journal.pone.0185569 28968427 PMC5624699

[30] Bezirtzoglou E. The intestinal microflora during the first weeks of life. Anaerobe. (1997) 3:173–7. 10.1006/anae.1997.0102 16887585

[31] Jiang HY. Diversity of Gut Microbiota Associated with Patients with Major Depressive Disorder. Hangzhou: Zhejiang University (2015).

[32] Deng Y Tang D Hou P Shen W Li H Wang T. Dysbiosis of gut microbiota in patients with esophageal cancer. Microb Pathog. (2020) 150:104709. 10.1016/j.micpath.2020.104709 33378710

[33] Chałupnik A Chilimoniuk Z Sobstyl A Dobosz M Borkowska A Wieteska M. Role of the gut microbiota in human health. J Educ Health Sport. (2020) 10:458–69. 10.12775/JEHS.2020.10.08.056

[34] Barrett E Kerr C Murphy K O'Sullivan O Ryan CA Dempsey EM. The individual-specific and diverse nature of the preterm infant microbiota. Arch Dis Child Fetal Neonatal Ed. (2013) 98:F334–40. 10.1136/archdischild-2012-303035 23303303

[35] Lin A Bik EM Costello EK Dethlefsen L Haque R Relman DA. Distinct distal gut microbiome diversity and composition in healthy children from Bangladesh and the United States. PLoS ONE. (2013) 8:e53838. 10.1371/journal.pone.0053838 23349750 PMC3551965

[36] Gabriel I Olejekb A Stencel-Gabriel K Wielgoś M. The influence of maternal vaginal flora on the intestinal colonization in newborns and 3-month- old infants. J Maternal Frtal Neonatal Med. (2017) 31:1448–53. 10.1080/14767058.2017.1319352 28420276

[37] Naseribafrouei A Hestad K Avershina E Sekelja M Linlkken A Wilson R. Correlation between the human fecal microbiota and depression. Neurogastroenterol Motil. (2014) 26:1155–62. 10.1111/nmo.12378 24888394

[38] Wu Y Luo Z Liu C. Variations in fecal microbial profiles of acute exacerbations and stable chronic obstructive pulmonary disease. Life Sci. (2021) 265:118738. 10.1016/j.lfs.2020.118738 33181175

[39] Hulin SJ Singh S Chapman MAS Allan A Langman MJS Eggo MC. Sulphide-induced energy deficiency in colonic cells is prevented by glucose but not by butyrate. Aliment Pharmacol Ther. (2002) 16:325–31. 10.1046/j.1365-2036.2002.01164.x 11860416

[40] Gibson G Macfarlane G Cummings J. Sulphate reducing bacteria and hydrogen metabolism in the human large intestine. Gut. (1993) 34:437–9. 10.1136/gut.34.4.437 8491386 PMC1374298

[41] Bisson-Boutelliez C Massin F Dumas D Miller N Lozniewski A. *Desulfovibrio* spp. survive within KB cells and modulate inflammatory responses. Mol Oral Microbiol. (2010) 25:226–35. 10.1111/j.2041-1014.2009.00550.x 20536750

[42] Chumpitazi BP Hoffman KL Smith DP McMeans AR Musaad S Versalovic J. Fructan-sensitive children with irritable bowel syndrome have distinct gut microbiome signatures. Aliment Pharmacol Therapeut. (2020) 53:499–509. 10.1111/apt.16204 33314183 PMC8281336

[43] Maa T Jina H Kwoka LY Suna Z Liongb MT Zhanga H. Probiotic consumption relieved human stress and anxiety symptoms possibly via modulating the neuroactive potential of the gut microbiota. Neurobiol Stress. (2021) 14:100294. 10.1016/j.ynstr.2021.100294 33511258 PMC7816019

[44] Qiu X Macchietto MG Liu X Lu Y Ma Y Guo H. Identification of gut microbiota and microbial metabolites regulated by an antimicrobial peptide lipocalin 2 in high fat diet-induced obesity. Int J Obes. (2021) 45:143–54. 10.1038/s41366-020-00712-2 33214705 PMC7755824

[45] Liu T Tao W Liang Q Tu WQ Xiao Y Chen L. Gut microbiota-related evidence provides new insights into the association between activating transcription factor 4 and development of salt-induced hypertension in mice. Front Cell Dev Biol. (2020) 8:585995. 10.3389/fcell.2020.585995 33282868 PMC7691383

[46] Jiang Z Shen K Shen Y. Zhu Futang Practice of Pediatrics. 8th ed. Beijing: People's Medical Publishing House (2015). p. 450–70.

[47] Carlson AL Xia K Azcarate-Peri MA Goldman BD Ahn M Styner MA. Infant gut microbiome associated with cognitive development. Biol Psychiatry. (2018) 83:148–59. 10.1016/j.biopsych.2017.06.021 28793975 PMC5724966

[48] Benítez-Páez A Gómez del Pugar EM López-Almela I Moya-Pérez A Codoñer-Franch P Sanz Y. Depletion of *Blautia* species in the microbiota of obese children relates to intestinal inflammation and metabolic phenotype worsening. mSystems. (2020) 5:e00209–19. 10.1128/mSystems.00857-19 32209719 PMC7093825

[49] Leylabadlo HE Ghotaslou R Feizabadi MM Farajnia S Moaddab SY Ganbarov K. The critical role of *Faecalibacterium prausnitzii* in human health: an overview. Microb Pathog. (2020) 149:104344. 10.1016/j.micpath.2020.104344 32534182

[50] De Filippis F Pasolli E Ercolini D. Newly explored *Faecalibacterium* diversity is connected to age, lifestyle, geography, and disease. Curr Biol. (2020) 30:4932–43.e4. 10.1016/j.cub.2020.09.063 33065016

[51] Al-Asmakh M Anuar F Zadjali F. Gut microbial communities modulating brain development and function. Gut Microb. (2012) 3:366–73. 10.4161/gmic.21287 22743758 PMC3463494

[52] Paharik AE Parlet CP Chung N Todd DA Rodriguez EI Van Dyke MJ. Coagulase-negative *Staphylococcal* strain prevents *Staphylococcus aureus* colonization and skin infection by blocking quorum sensing. Cell Host Microbe. 22:746–56.e5. 10.1016/j.chom.2017.11.001 29199097 PMC5897044

[53] O'Sullivan A Farver M Smilowitz JT. The inflfluence of early infant-feeding practices on the intestinal microbiome and body composition in infants. Nutr Metab Insights. (2016) 2015(Suppl. 1):1–9. 10.4137/NMI.S41125 26715853 PMC4686345

[54] van Best N Hornef MW Savelkoul PHM Penders J. On the origin of species: factors shaping the establishment of infant's gut microbiota. Birth Defects Res C Embryo Today. (2015) 105:240–51. 10.1002/bdrc.21113 26607554

[55] Bäckhed F Roswall J Peng YQ Feng Q Jia H Kovatcheva-Datchary P. Dynamics and stabilization of the human gut microbiome during the first year of life. Cell Host Microbe. (2015) 17:690–703. 10.1016/j.chom.2015.04.004 25974306

[56] Schwarzenberg SJ Georgieff MK. Advocacy for improving nutrition in the first 1000 days to support childhood development and adult health. Pediatrics. (2018) 141:e20173716. 10.1542/peds.2017-3716 29358479

[57] Williams JE Carrothers JM Lackey KA Beatty NF Brooker SL Peterson HK. Strong multivariate relations exist among milk, oral, and fecal microbiomes in mother-infant dyads during the first six months postpartum. J Nutr. (2019) 149:902–14. 10.1093/jn/nxy299 31063198 PMC6543206

[58] Yang B Chen Y Stanton C Ross RP Lee YK Zhao J. *Bifidobacterium* and *Lactobacillus* composition at species level and gut microbiota diversity in infants before 6 weeks. Int J Mol Sci. (2019) 20:3306. 10.3390/ijms20133306 31284413 PMC6650860

[59] Bokulich NA Chung J Battaglia T Henderson N Jay M Li H. Antibiotics, birth mode, and diet shape microbiome maturation during early life. Sci Transl Med. (2016) 8:343ra82. 10.1126/scitranslmed.aad7121 27306664 PMC5308924

[60] Thompson AL Monteagudo-Mera A Cadenas MB Lampl ML Azcarate-Peril MA. Milk- and solid-feeding practices and daycare attendance are associated with differences in bacterial diversity, predominant communities, and metabolic and immune function of the infant gut microbiome. Front Cell Infect Microbiol. (2015) 5:3. 10.3389/fcimb.2015.00003 25705611 PMC4318912

[61] Baumann-Dudenhoeffer AM D'Souza AW Tarr PI Warner BB Dantas G. Infant diet and maternal gestational weight gain predict early metabolic maturation of gut microbiomes. Nat Med. (2018) 24:1822–9. 10.1038/s41591-018-0216-2 30374198 PMC6294307

[62] Madan JC Hoen AG Lundgren SN Farzan SF Cottingham KL Morrison HG. Association of cesarean delivery and formula supplementation with the intestinal microbiome of 6-week-old infants. JAMA Pediatr. (2016) 170:212–9. 10.1001/jamapediatrics.2015.3732 26752321 PMC4783194

[63] Dominguez-Bello MG Costello EK Contreras M Magris M Hidalgo G Fierer N. Delivery mode shapes the acquisition and structure of the initial microbiota across multiple body habitats in newborns. Proc Natl Acad Sci USA. (2010) 107:11971–5. 10.1073/pnas.1002601107 20566857 PMC2900693

[64] Gosalbes MJ Llop S Vallès Y Moya A Ballester F Francino MP. Meconium microbiota types dominated by lactic acid or enteric bacteria are differentially associated with maternal eczema and respiratory problems in infants. Clin Exp Allergy. (2013) 43:198–211. 10.1111/cea.12063 23331561

[65] Limbana T Khan F Eskander N. Gut microbiome and depression: how microbes affect the way we think. Cureus. (2020) 12:e9966. 10.7759/cureus.9966 32983670 PMC7510518

