# Supplementary material for: Evolution of Intestinal Microbiota of Asphyxiated Neonates Within 1 Week and Its Relationship With Neural Development at 6 Months
Source: Front Pediatr. 2021 Aug 23;9:690339. doi: 10.3389/fped.2021.690339 (PMC8419515; doi:10.3389/fped.2021.690339)
Supplement: Supplementary file 1 [file Data_Sheet_1.docx]

Table1 comparison of Alpha diversity of fecal flora in the study group at 1d,3d and 5d after birth

| days | chao1 | simpson | Observed_species | ACE | Shannon |
| --- | --- | --- | --- | --- | --- |
| D1 | 345.68 | 0.57±0.26 | 269.0 | 378.79 | 2.36 |
| D3 | 238.22 | 0.42±0.22 | 180 | 276.25 | 1.626 |
| D5 | 181.84 | 0.42±0.24 | 121 | 195.39 | 1.31 |
| X^2^/F | 8.0 | 5.09 | 11.09 | 7.03 | 4.41 |
| p | 0.018 | 0.008 | 0.004 | 0.03 | 0.11 |
| D1 | 345.68 | 0.57±0.26 | 269.0 | 378.79 |  |
| D3 | 238.22 | 0.42±0.22 | 180 | 276.25 |  |
| t/z | -2.93 | -3.12 | -3.2 | -2.87 |  |
| p | 0.003 | 0.003 | 0.001 | 0.004 |  |
| D3 | 238.22 | 0.42±0.22 | 180 | 276.25 |  |
| D5 | 181.84 | 0.42±0.24 | 121 | 195.39 |  |
| t/z | -0.86 | -1.74 | -1.23 | -0.67 |  |
| p | 0.389 | 0.092 | 0.221 | 0.505 |  |

Table2 Comparison of Alpha diversity between the study group and control group at 1d,3d and 5d

| days | Alpha index | Study group | Control group | t/z | p |
| --- | --- | --- | --- | --- | --- |
| D1 | chao1 | 345.68 | 207.08 | -2.57 | 0.01 |
|  | simpson | 0.57±0.26 | 0.41±0.26 | 2.62 | 0.011 |
|  | Observed_species | 269.00 | 154.50 | -2.69 | 0.007 |
|  | ACE | 378.79 | 203.21 | -2.57 | 0.01 |
|  | Shannon | 2.36 | 1.43 | -2.88 | 0.004 |
| D3 | chao1 | 238.22 | 206.53 | -1.199 | 0.231 |
|  | simpson | 0.42±0.22 | 0.39±0.21 | 0.639 | 0.525 |
|  | Observed_species | 180.00 | 134.50 | -1.69 | 0.091 |
|  | ACE | 276.25 | 226.58 | -1.189 | 0.235 |
|  | Shannon | 1.63 | 1.3 | -0.972 | 0.331 |
| D5 | chao1 | 181.84 | 138.48 | 1.307 | 0.191 |
|  | simpson | 0.42±0.24 | 0.42±0.2 | -0.092 | 0.927 |
|  | Observed_species | 121 | 86.5 | -0.948 | 0.325 |
|  | ACE | 195.39 | 154.02 | -0.967 | 0.333 |
|  | Shannon | 1.31 | 1.41 | -0.153 | 0.879 |


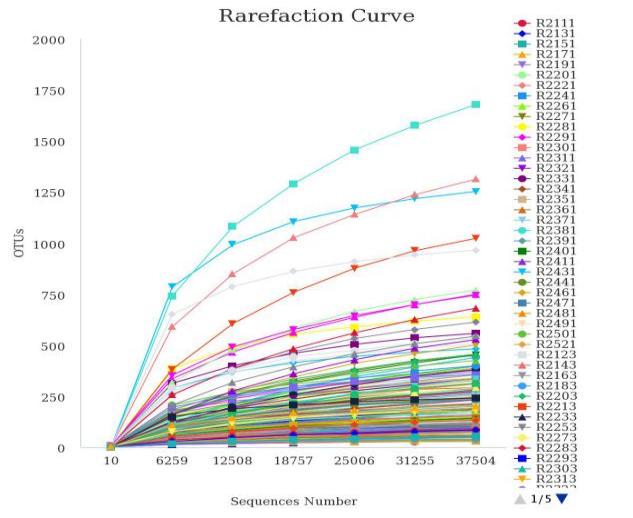


Figure a

Figure a. The Rarefaction Curve of each sample of the two groups.


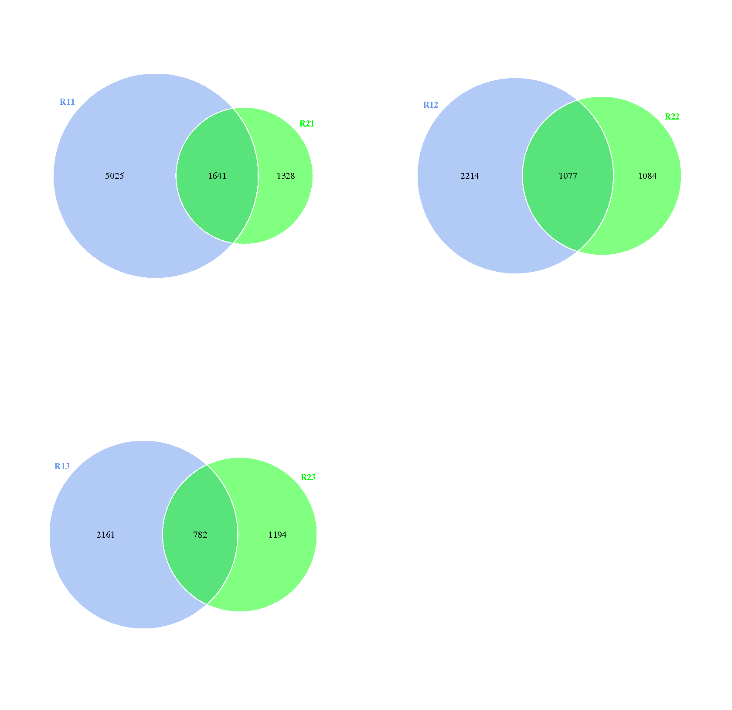
 Figure b

Figure b, Venn picture of the two groups on day 1,3,5 of life after birth.


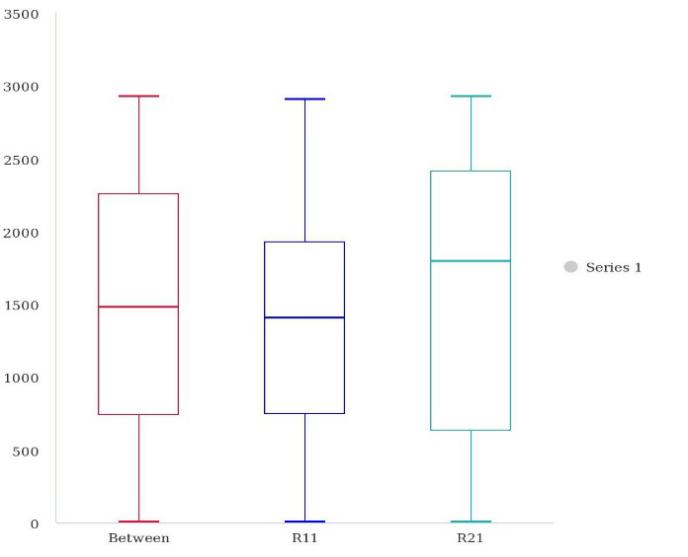

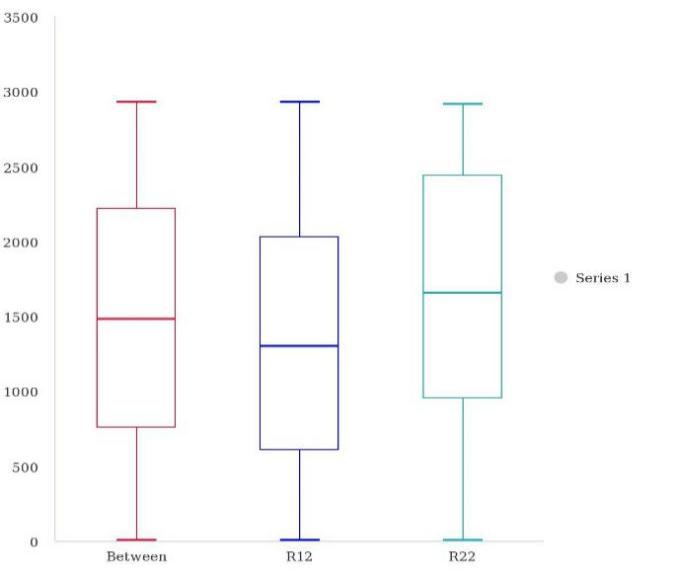


Figure c1 Figure c2


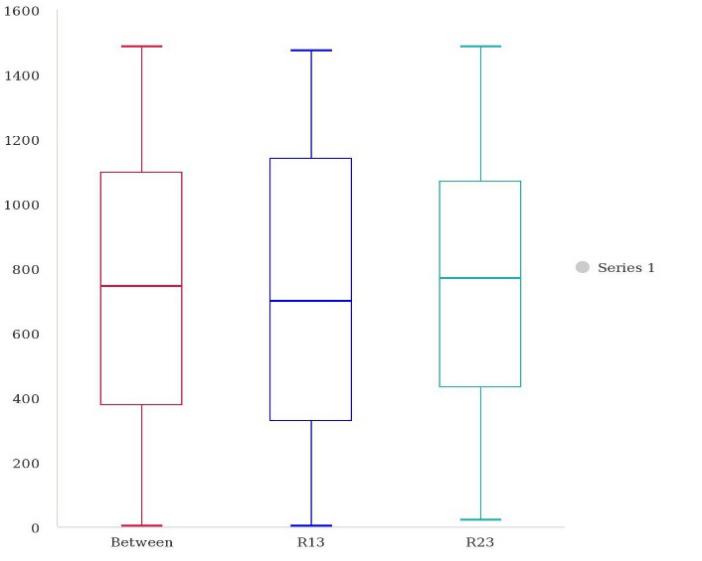

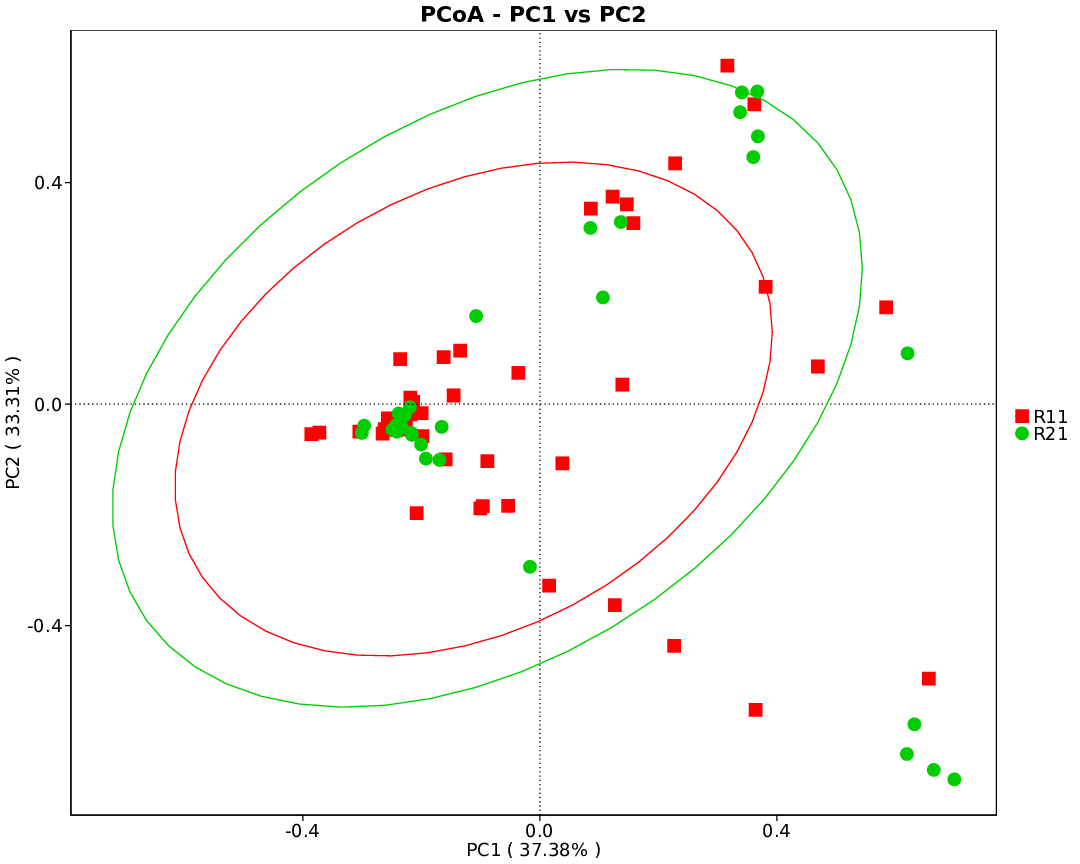


Figure c3 Figure c4


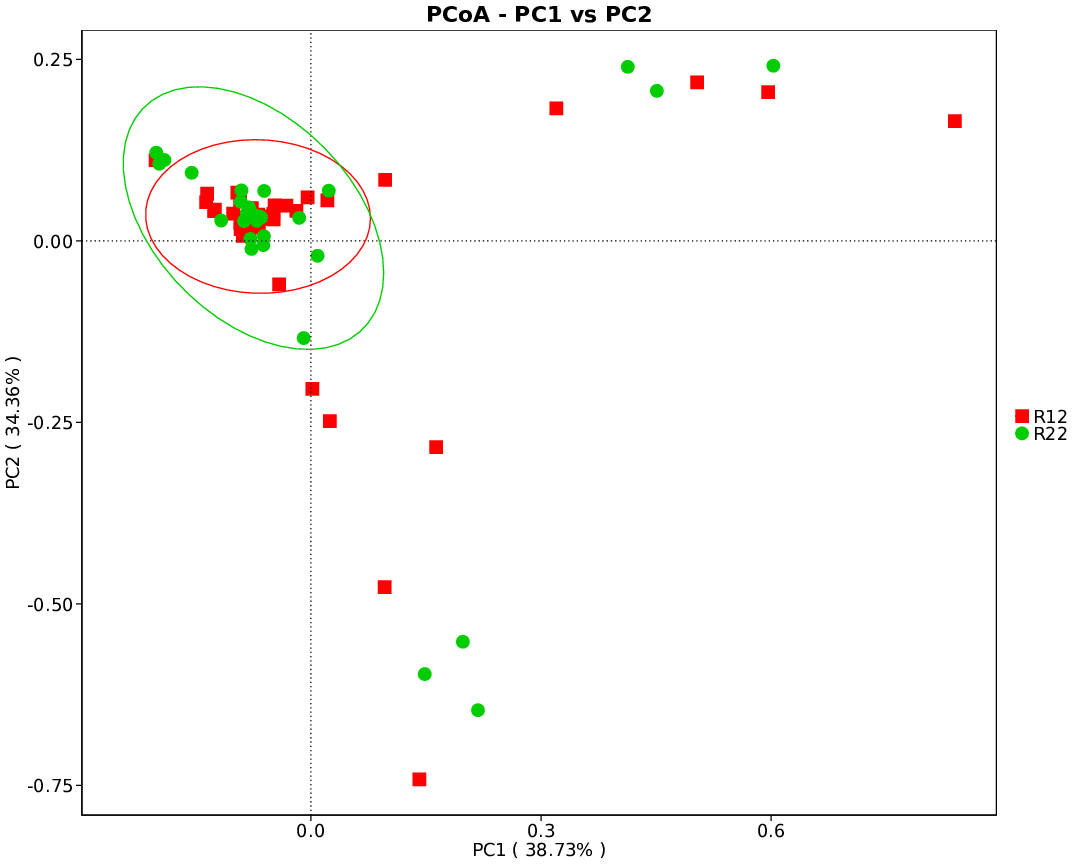

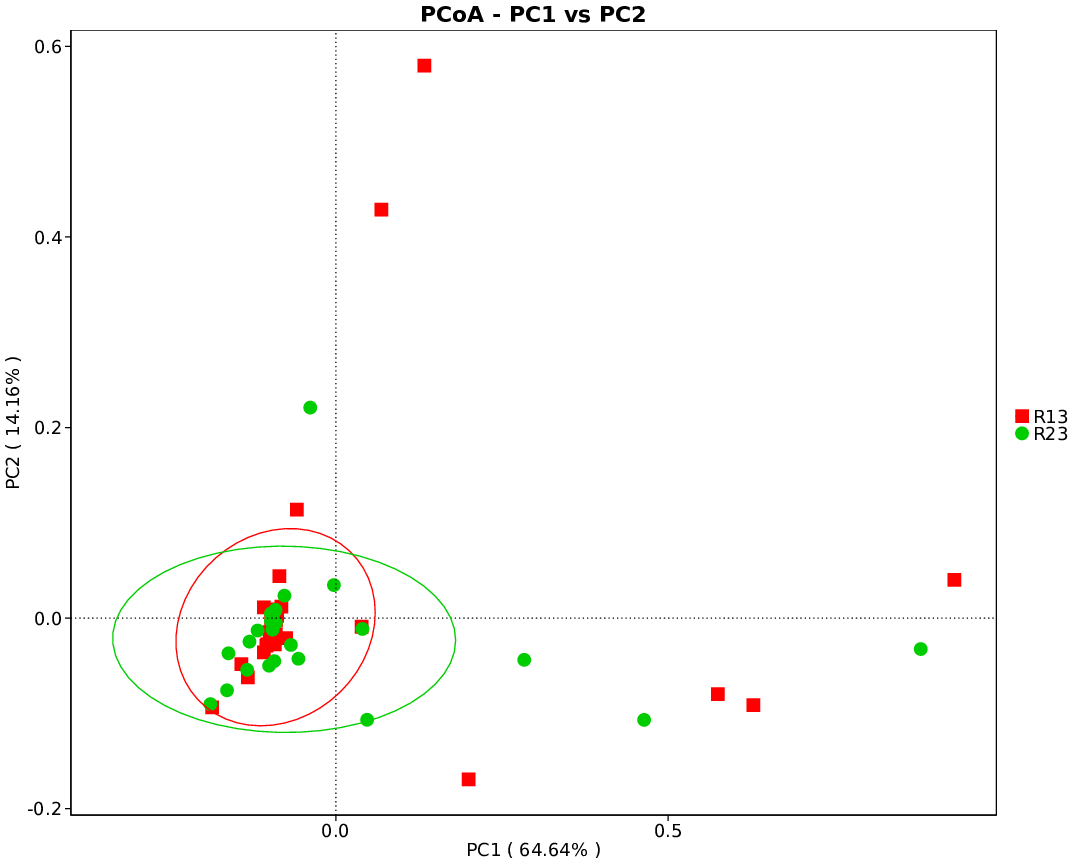


Figure c5 Figure c6

Figure c. microbial differences between the study group and control group on days 1,3 and 5. c1, microbial differences between the two group on day 1; c2, microbial differences between the two group on day 3; c3, microbial differences between the two group on day 5;c4, PCoA analysis between the two group on day 1; c5, PCoA analysis between the two group on day 3; c6, PCoA analysis between the two group on day 5; R11, gut flora of the study group on day 1; R21, gut flora of the control group on day 1; R12, gut flora of the study group on day 3; R22, gut flora of the control group on day 3; R13, gut flora of the study group on day 5; R23, gut flora of the control group on day 5.

Table 3, PCoA analysis of the study group and control group on days 1,3 and 5 after birth.

| days | R-value | P-value |
| --- | --- | --- |
| D1 | 0.033 | 0.11 |
| D3 | 0.031 | 0.13 |
| D5 | 0.0055 | 0.33 |


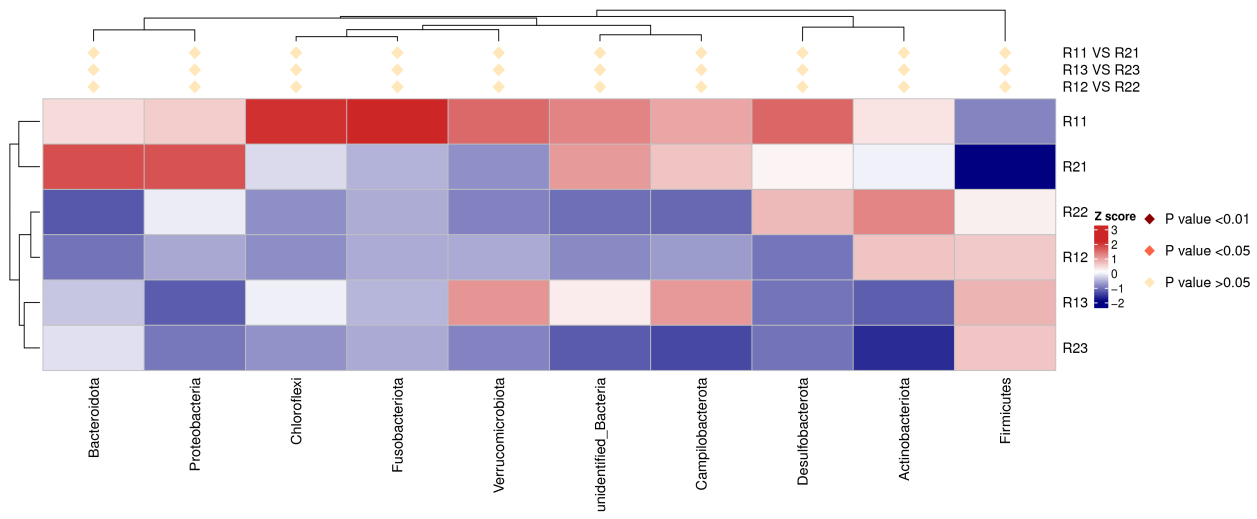


Figure d1


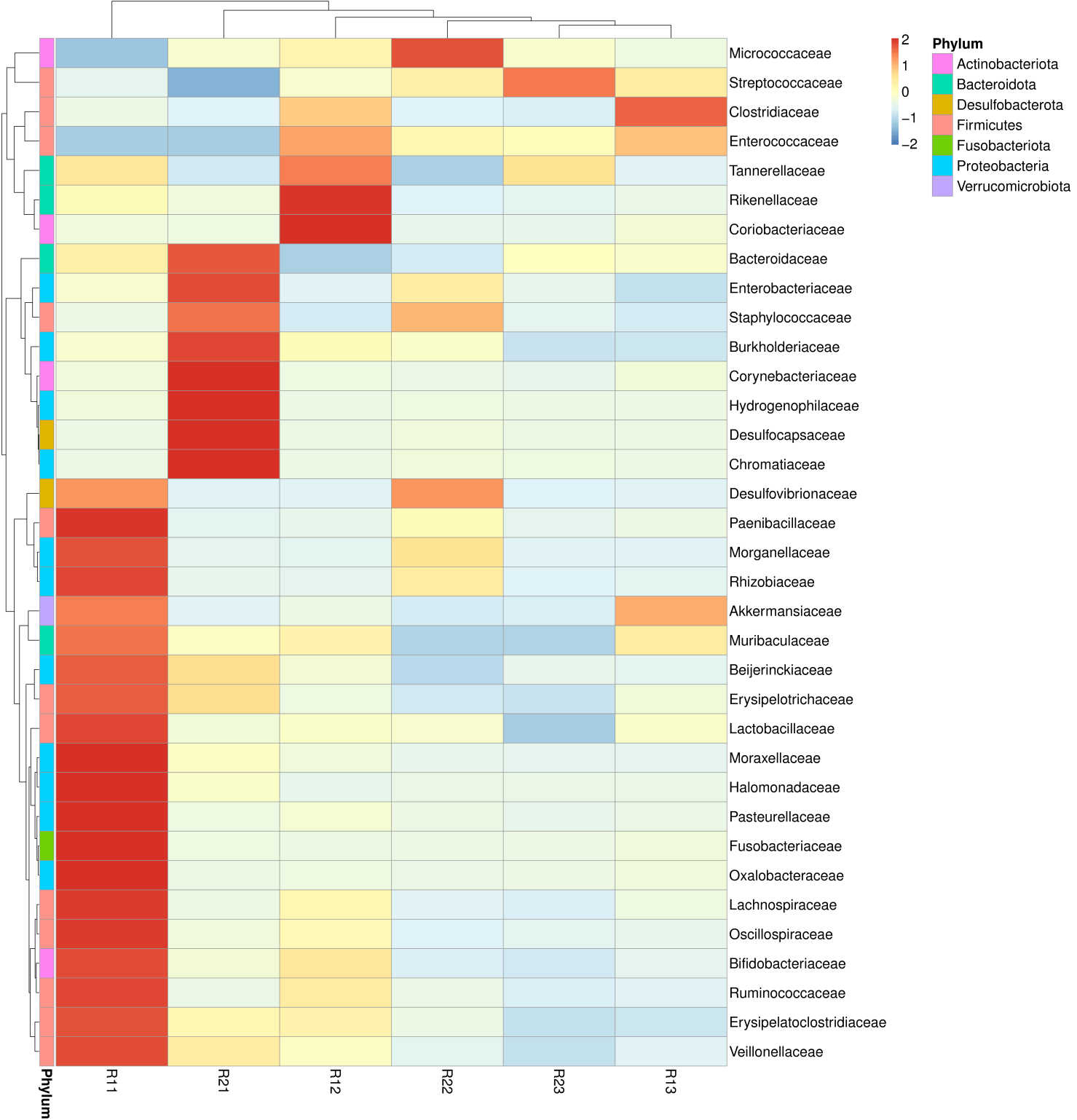


Figure d2

Figure d. d1, Results of heatmap analysis of species with significant difference in phylum level; d2, The heatmap of taxonomy abundance in each group at Phylum level ; R11, gut flora of the study group on day 1; R21, gut flora of the control group on day 1; R12, gut flora of the study group on day 3; R22, gut flora of the control group on day 3; R13, gut flora of the study group on day 5; R23, gut flora of the control group on day 5.


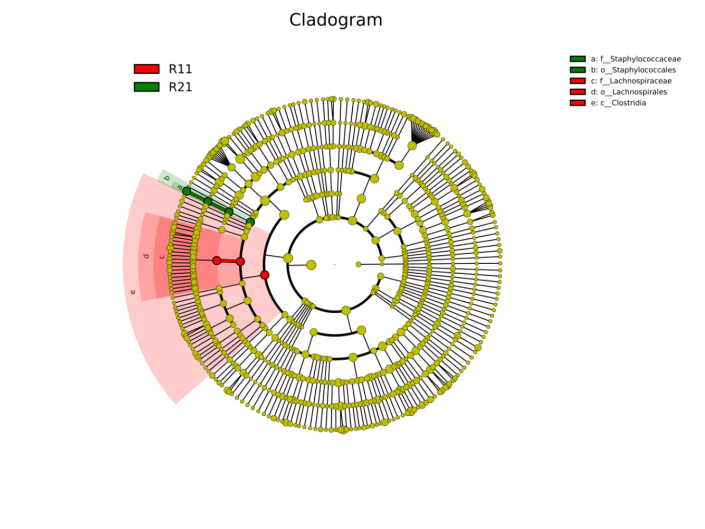

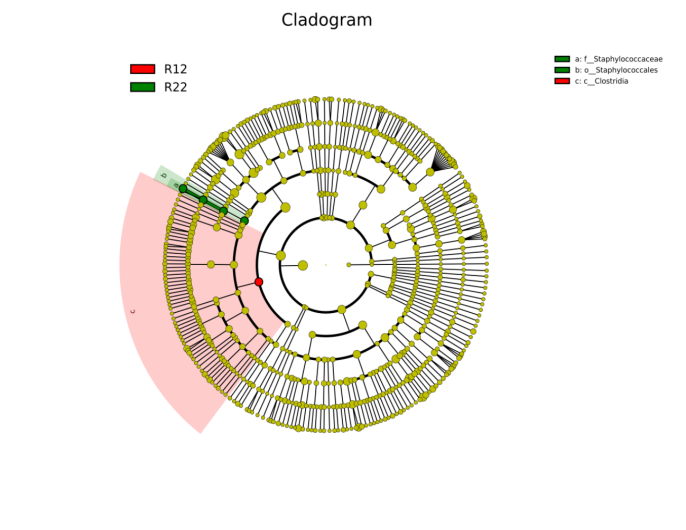


Figure e1 Figure e2

Figure e, e1: cladogram of the two groups on day 1 of life; e2:cladogram of the two groups on day 3 of life.

Table4 Abbreviation list

| Abbreviation | Full name |
| --- | --- |
| rDNA | ribosome Deoxyribonucleic Acid |
| ASQ-3 | Ages and Stages Questionnaires-3 |
| NMDS | Non-Metric Multi-Dimensional Scaling |
| LEfSe | Linear Discriminant Analysis Effect Size |
| ADHD | Attention Deficit Hyperactivity Disorder |
| ASD | Autism Spectrum Disorders |
| OTUs | Operational Taxonomic Units |
| PCoA | Principal Co-ordinates Analysis |
| ACE | abundance-based coverage estimator |
| PCR | poly-merase chain reaction |
| SPSS | Statistical Package for Social Sciences |

Table 5.Comparison of Alpha diversity in the mixed feeding asphyxial neonates at 1d,3d and 5d after birth

| days | chao1 | simpson | Observed_species | ACE | Shannon |
| --- | --- | --- | --- | --- | --- |
| D1 | 194.4 | 0.62±0.26 | 164.00 | 203.58 | 2.63±1.34 |
| D3 | 308.77±209.25 | 0.44±0.19 | 153.00 | 329.63±222.81 | 1.63±0.73 |
| D5 | 183.77 | 0.49±0.22 | 131.00 | 195.39 | 1.59±0.77 |
| X^2^ | 3.73 | 4.93 | 5.20 | 2.8 | 5.20 |
| p | 0.16 | 0.085 | 0.074 | 0.25 | 0.074 |

Table 6.Comparison of Alpha diversity in formula feeding asphyxial neonates at 1d,3d and 5d after birth.

| days | chao1 | simpson | Observed_species | ACE | Shannon |
| --- | --- | --- | --- | --- | --- |
| D1 | 464.02 | 0.53±0.27 | 333.5 | 502.84 | 2.40±1.40 |
| D3 | 243.75 | 0.41±0.24 | 181.00 | 276.54 | 1.39 |
| D5 | 153.53 | 0.35±0.24 | 102.00 | 172.34 | 0.89 |
| X^2^ | 6.00 | 3.38 | 7.63 | 4.63 | 6.89 |
| p | 0.05 | 0.19 | 0.022 | 0.099 | 0.032 |
| D1 |  |  | 333.5 |  | 2.40±1.40 |
| D3 |  |  | 181.00 |  | 1.39 |
| t/z |  |  | -3.36 |  | -2.18 |
| p |  |  | 0.001 |  | 0.029 |
| D3 |  |  | 181.00 |  | 1.39 |
| D5 |  |  | 102.00 |  | 0.89 |
| t/z |  |  | -1.28 |  | -1.17 |
| p |  |  | 0.20 |  | 0.244 |

Table 7. Comparison of Alpha diversity between mixed feeding asphyxiated neonates and formula feeding at 1d, 3d and 5d.

| days | Alpha index | Formula+breast milk | Formula | t/z | p |
| --- | --- | --- | --- | --- | --- |
| D1 | chao1 | 194.40 | 464.02 | -2.14 | 0.033 |
|  | simpson | 0.62±0.26 | 0.53±0.27 | -1.13 | 0.27 |
|  | Observed_species | 164.00 | 333.50 | -2.29 | 0.022 |
|  | ACE | 203.58 | 502.84 | -2.21 | 0.027 |
|  | Shannon | 2.63±1.34 | 2.40±1.40 | -0.57 | 0.58 |
| D3 | chao1 | 308.77±209.25 | 243.75 | -0.30 | 0.77 |
|  | simpson | 0.44±0.19 | 0.41±0.24 | -0.35 | 0.73 |
|  | Observed_species | 153.00 | 181.00 | -0.23 | 0.82 |
|  | ACE | 329.63±222.81 | 276.54 | -0.18 | 0.85 |
|  | Shannon | 1.63±0.73 | 1.39 | -0.67 | 0.51 |
| D5 | chao1 | 183.77 | 153.53 | -0.04 | 0.97 |
|  | simpson | 0.49±0.22 | 0.35±0.24 | 1.62 | 0.12 |
|  | Observed_species | 131.00 | 102.00 | -0.34 | 0.74 |
|  | ACE | 195.39 | 172.34 | -0.16 | 0.87 |
|  | Shannon | 1.59±0.77 | 0.89 | -1.42 | 0.16 |

Table 8. PCoA analysis of the two feeding type.

| days | R-value | P-value |
| --- | --- | --- |
| D1 | -0.0096 | 0.55 |
| D3 | -0.032 | 0.82 |
| D5 | 0.12 | 0.008 |

Table 9. intestinal microbiota analysis of the two different feeding types at 1d,3d,5d.

| days | Taxonomy | Formula+breast milk | Formula | p |
| --- | --- | --- | --- | --- |
| D1 | Clostridium_sensu_stricto_1 | 5.33×10^-5^ | 6.67×10^-4^ | 0.009 |
|  | Parabacteroides | 3.07×10^-3^ | 3.73×10^-4^ | 0.027 |
| D3 | Ralstonia | 1.07×10^-4^ | 7.60×10^-4^ | 0.038 |
|  | Clostridium_sensu_stricto_1 | 5.33×10-^5^ | 2.67×10^-4^ | 0.015 |
|  | Escherichia-Shigella | 5.07×10^-4^ | 1.45×10^-3^ | 0.022 |
| D5 | Clostridium_sensu_stricto_1 | 2.67×10^-5^ | 4.40×10^-4^ | 0.003 |
|  | Streptococcus | 0.31 | 0.099 | 0.009 |
|  | Faecalibacterium | 0.00 | 9.33×10^-5^ | 0.002 |

**
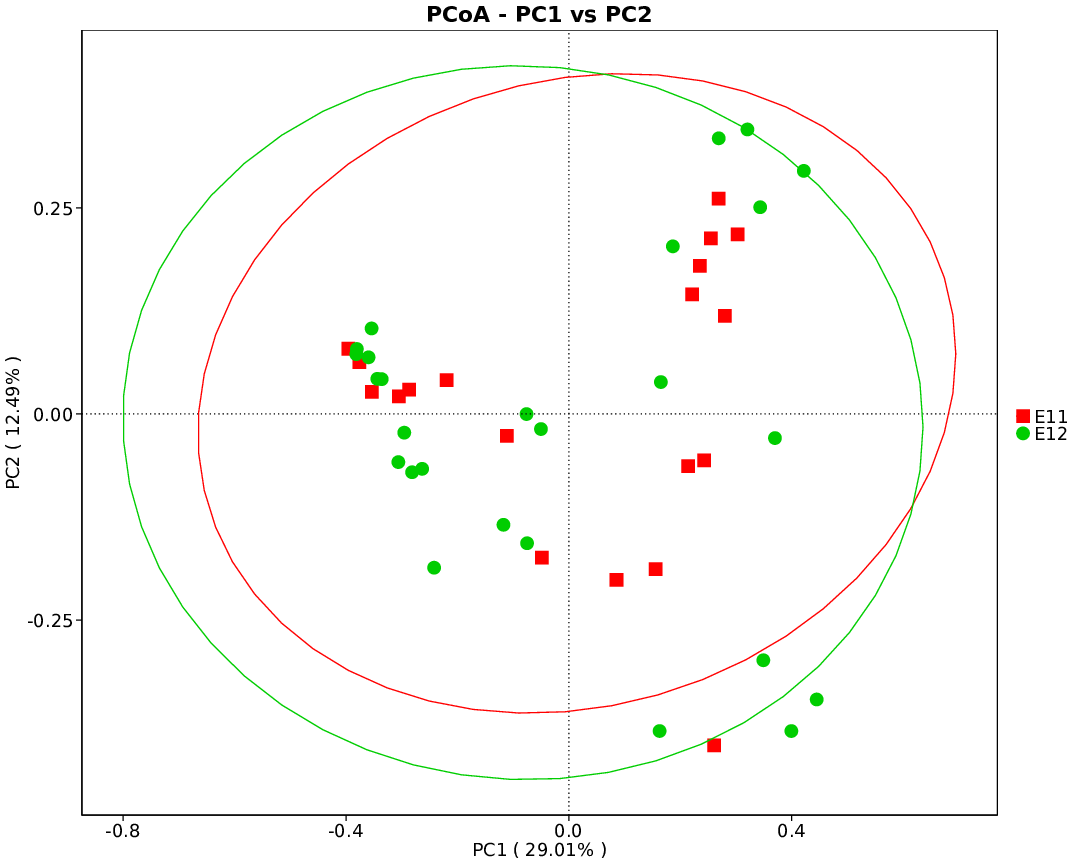

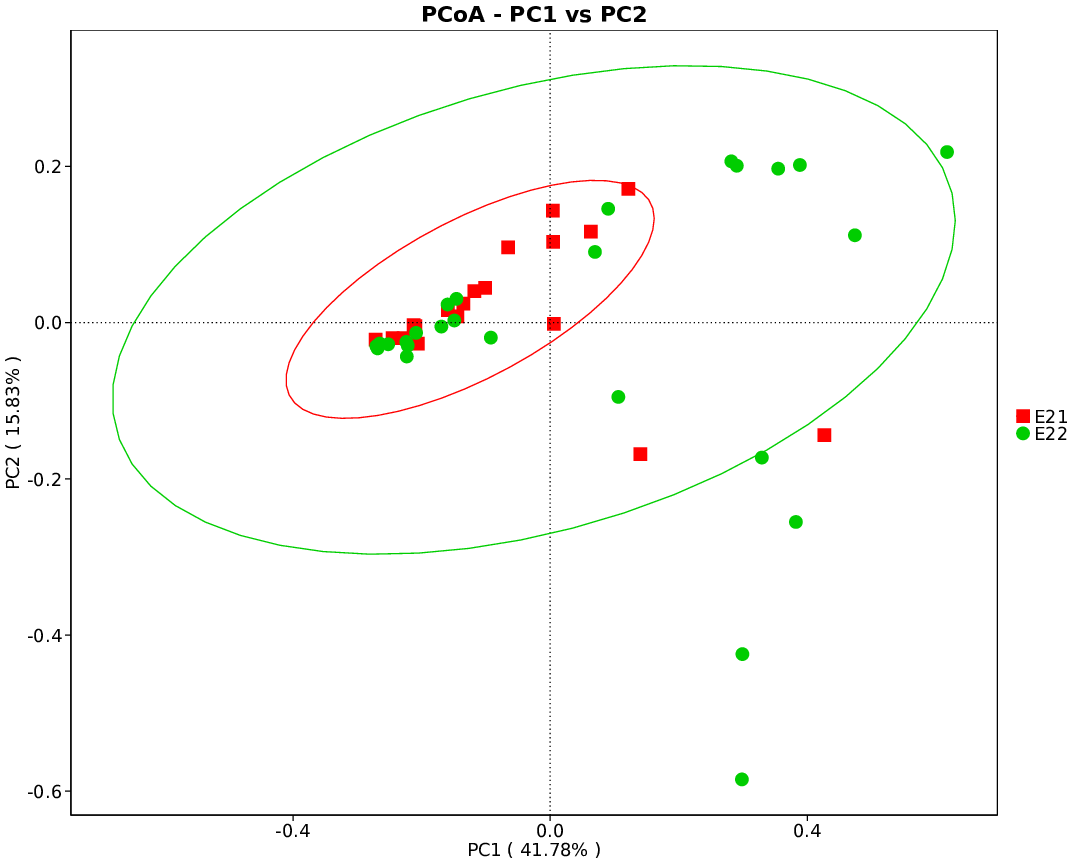
**

Figure f1 Figure f2

**
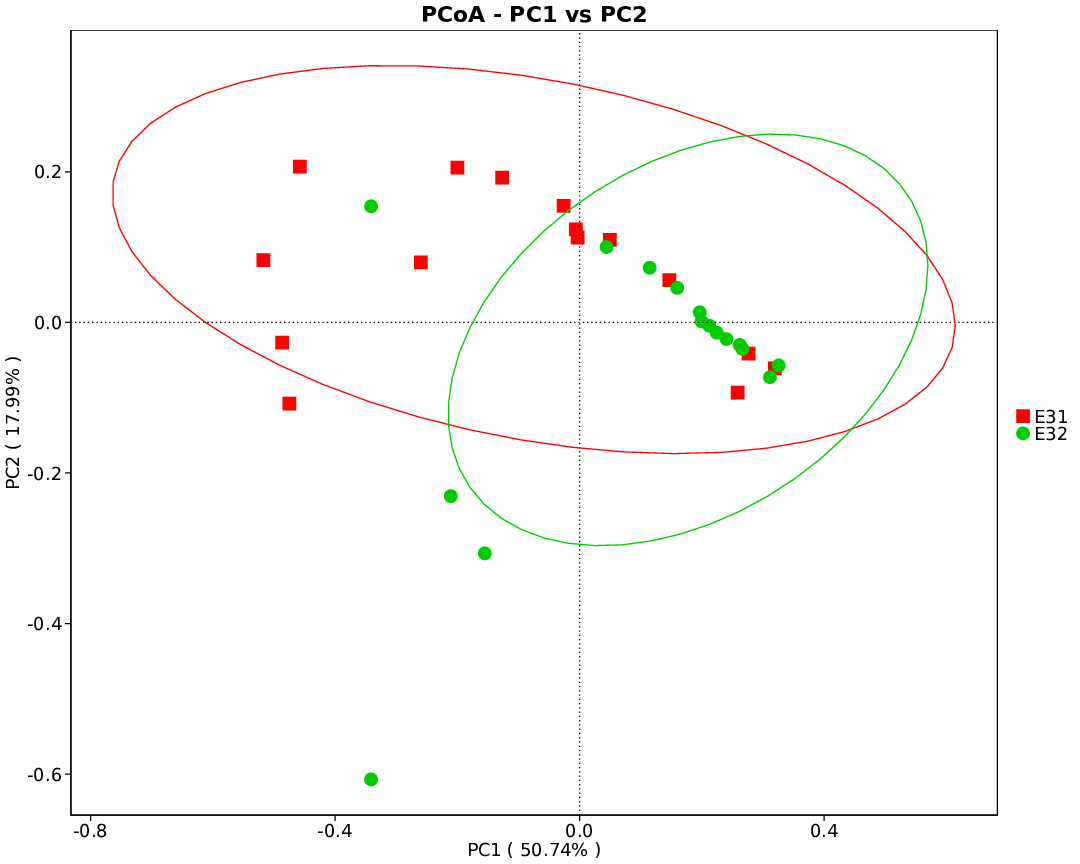
**

Figure f3

Figure f, f1.PCoA analysis between mixed feeding asphyxiated neonates and formula feeding at 1d;f2.PCoA analysis between mixed feeding asphyxiated neonates and formula feeding at 3d;f3.PCoA analysis between mixed feeding asphyxiated neonates and formula feeding at 5d.E11.gut flora of mixed feeding asphyxiated neonates at 1d;E12.gut flora of formula feeding asphyxiated neonates at 1d;E21.gut flora of mixed feeding asphyxiated neonates at 3d;E22.gut flora of formula feeding asphyxiated neonates at 3d;E31.gut flora of mixed feeding asphyxiated neonates at 5d;E32.gut flora of formula feeding asphyxiated neonates at 5d.


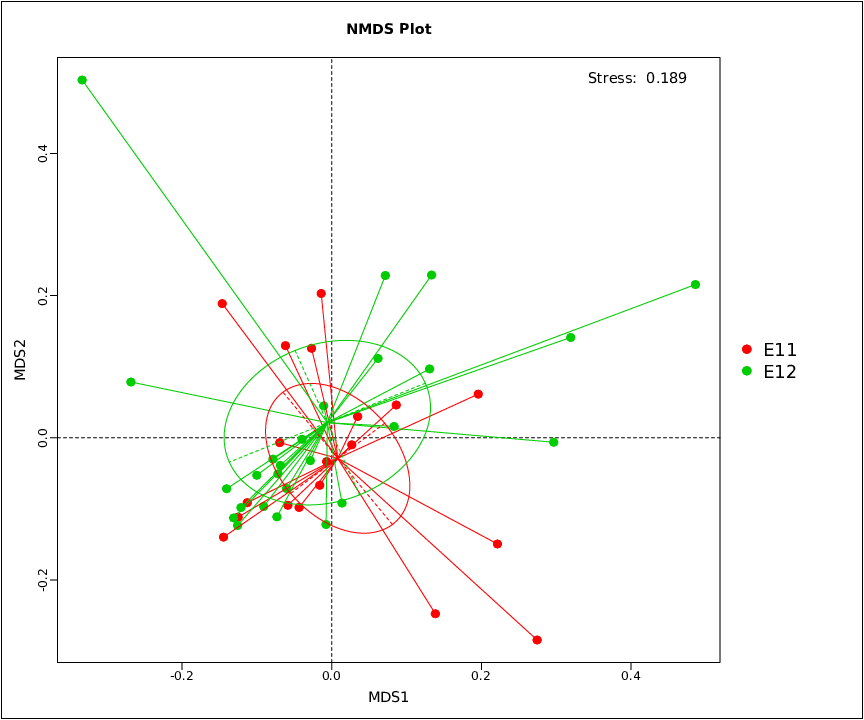

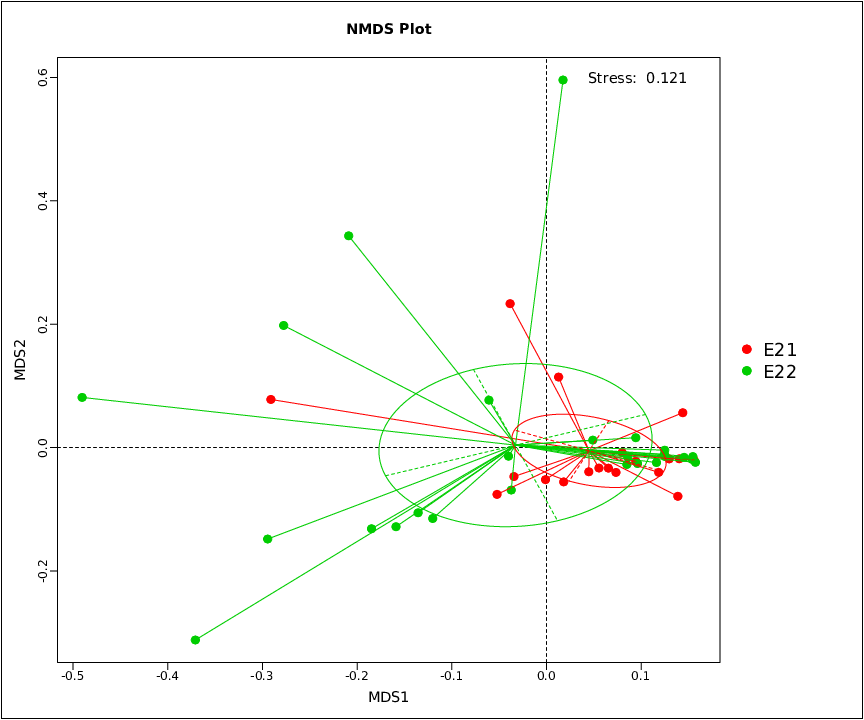


Figure g1 Figure g2


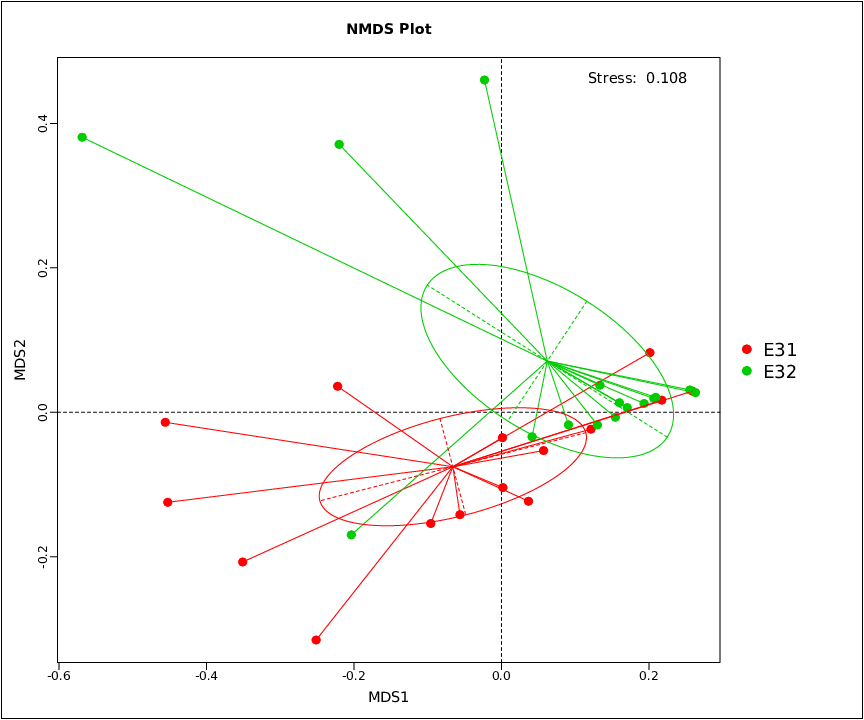


Figure g3

Figure g, g1.NMDS analysis between mixed feeding asphyxiated neonates and formula feeding at 1d;g2.NMDS analysis between mixed feeding asphyxiated neonates and formula feeding at 3d;g3.NMDS analysis between mixed feeding asphyxiated neonates and formula feeding at 5d.


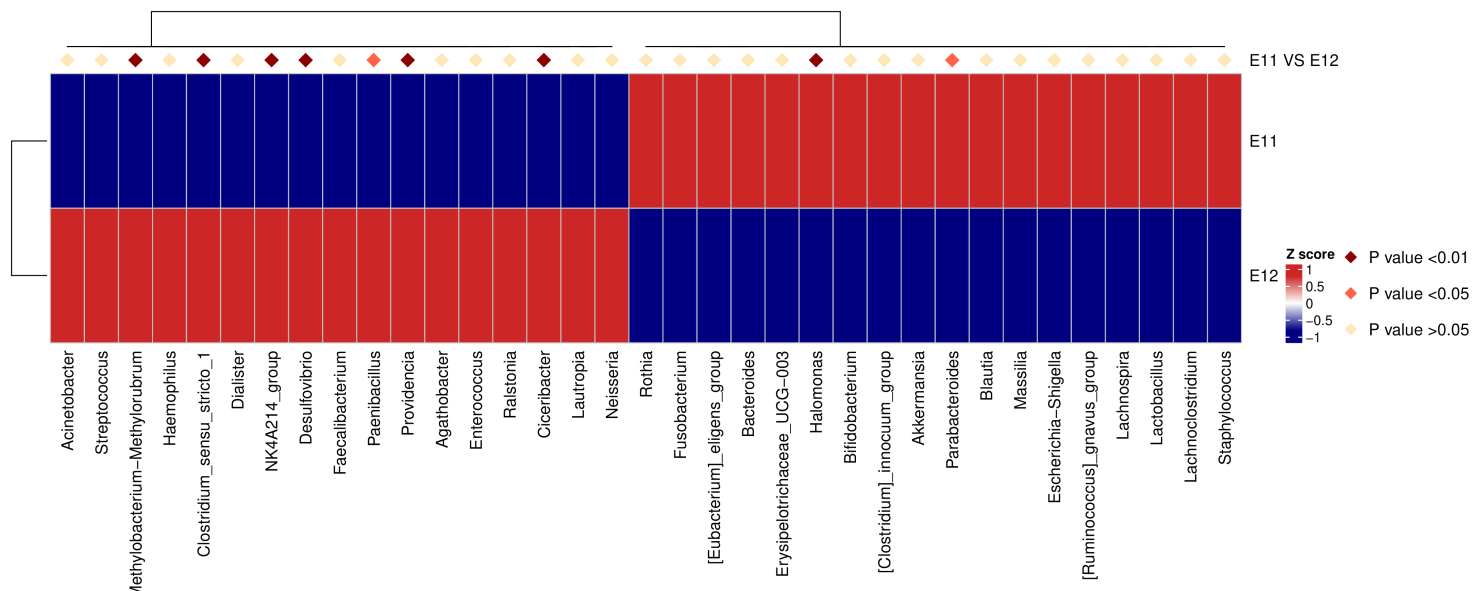


Figure h1


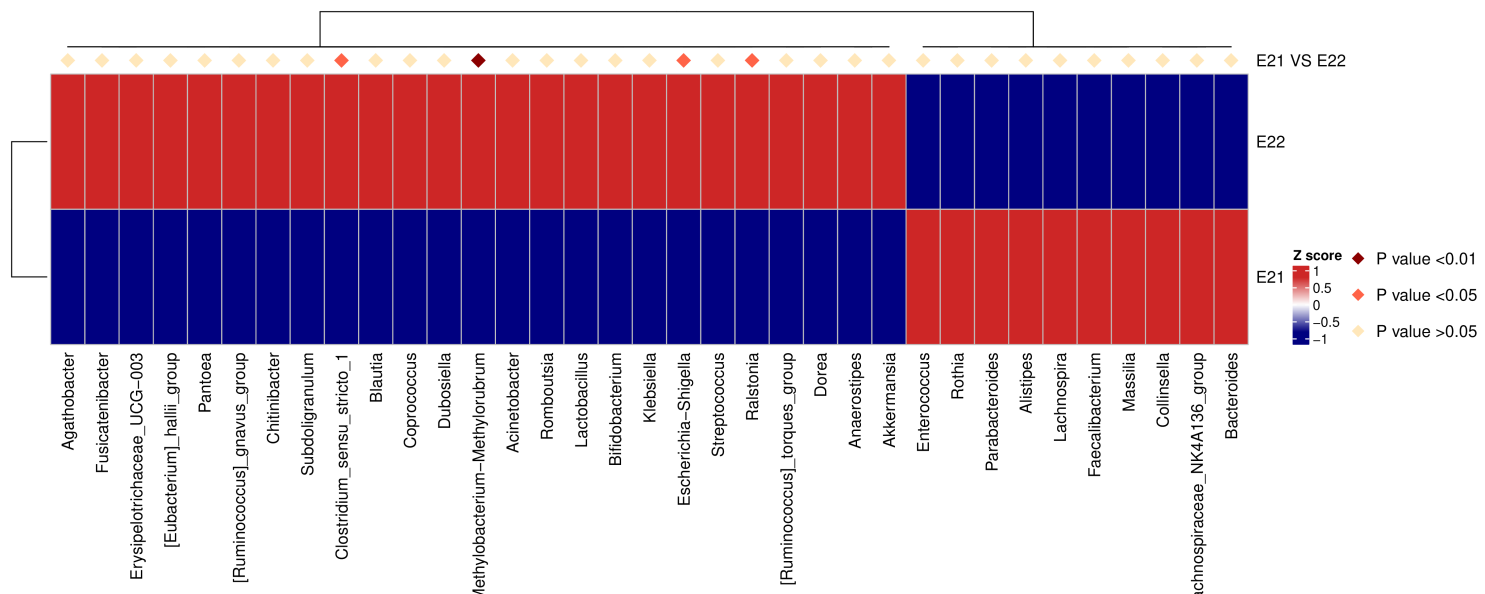


Figure h2


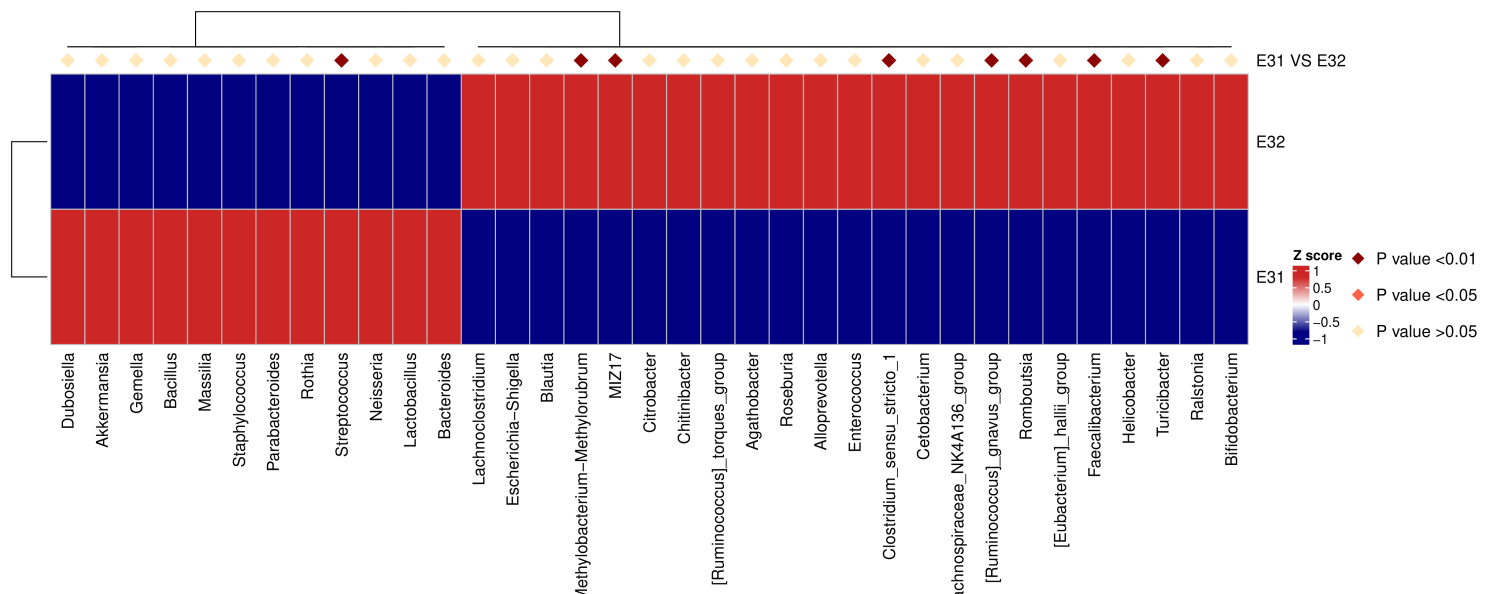


Figure h3

Figure h,h1.Results of heatmap analysis of species with significant difference in genus level at 1d;h2.Results of heatmap analysis of species with significant difference in genus level at 3d;h3.Results of heatmap analysis of species with significant difference in genus level at 5d.


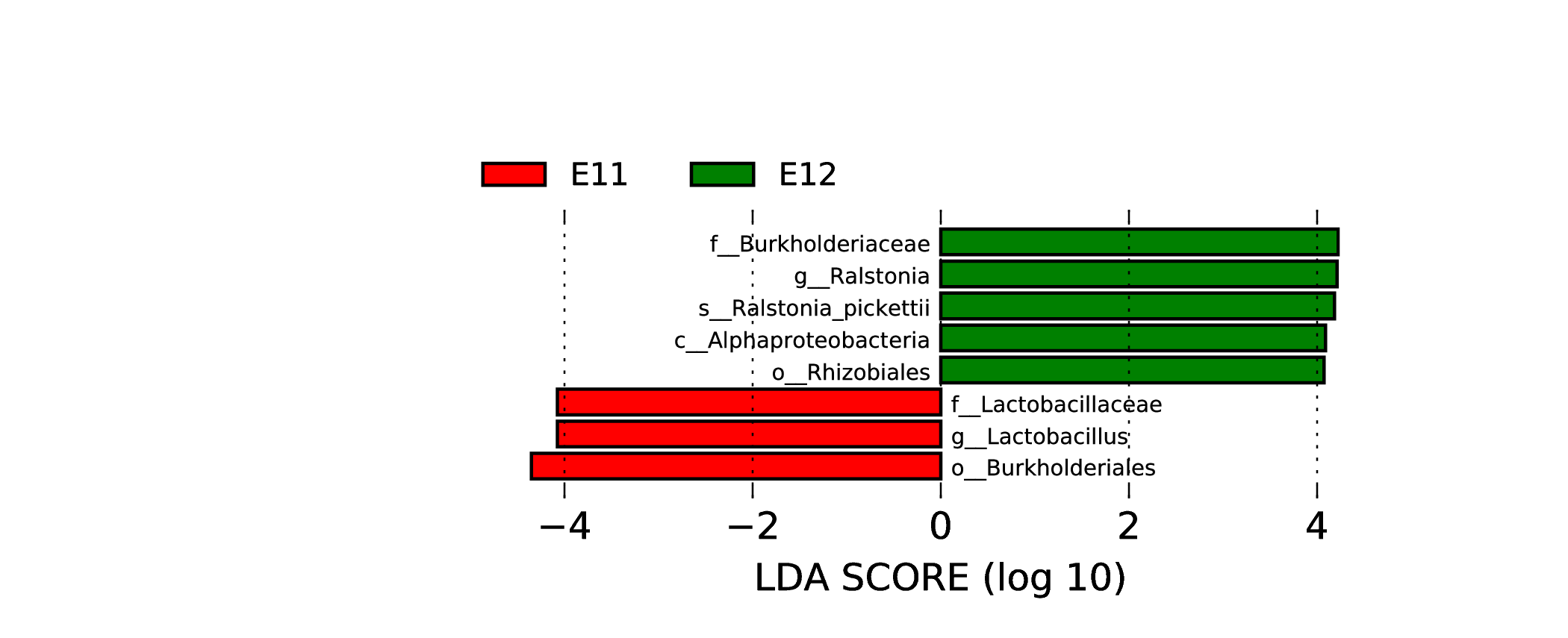

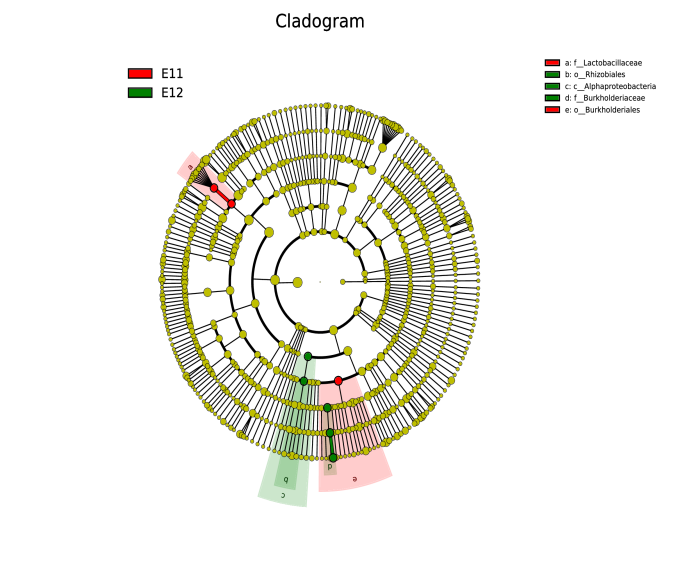


Figure j1 Figure j2


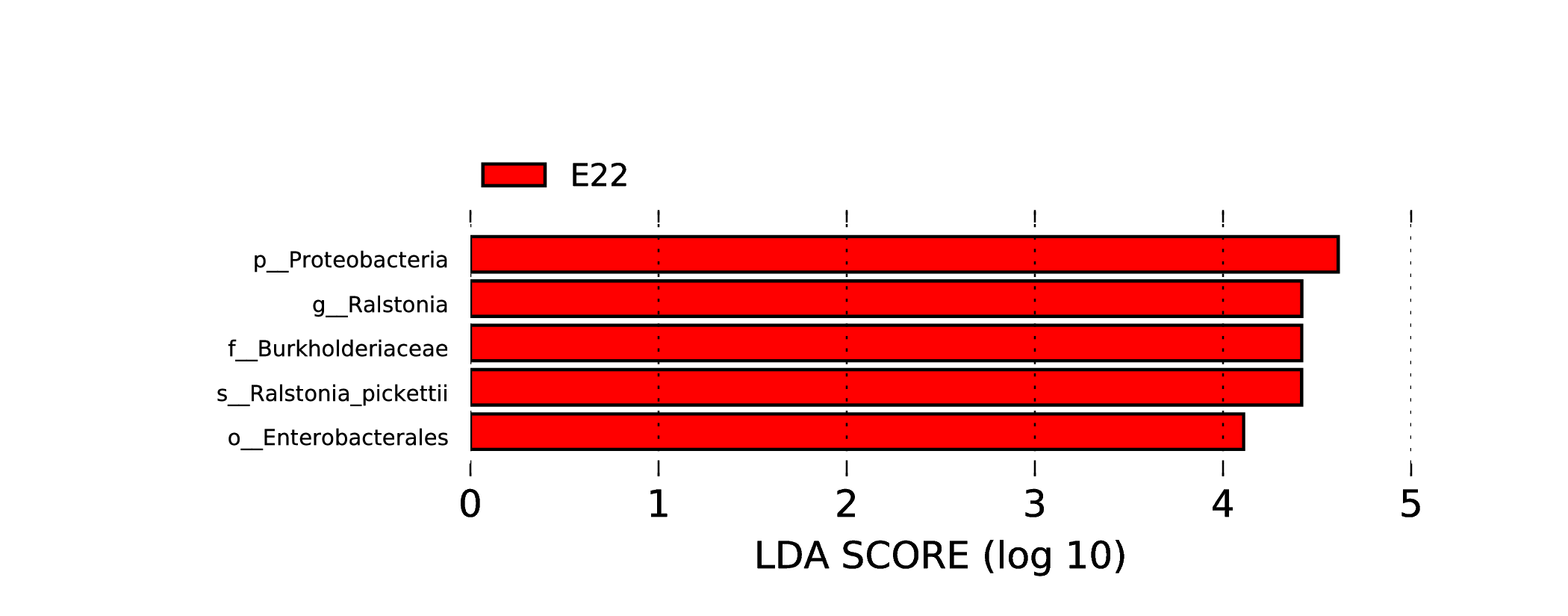

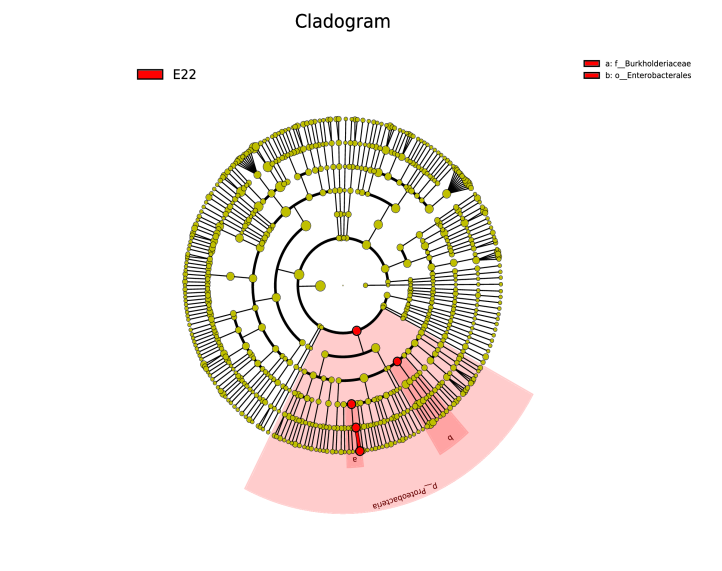


Figure j3 Figure j4


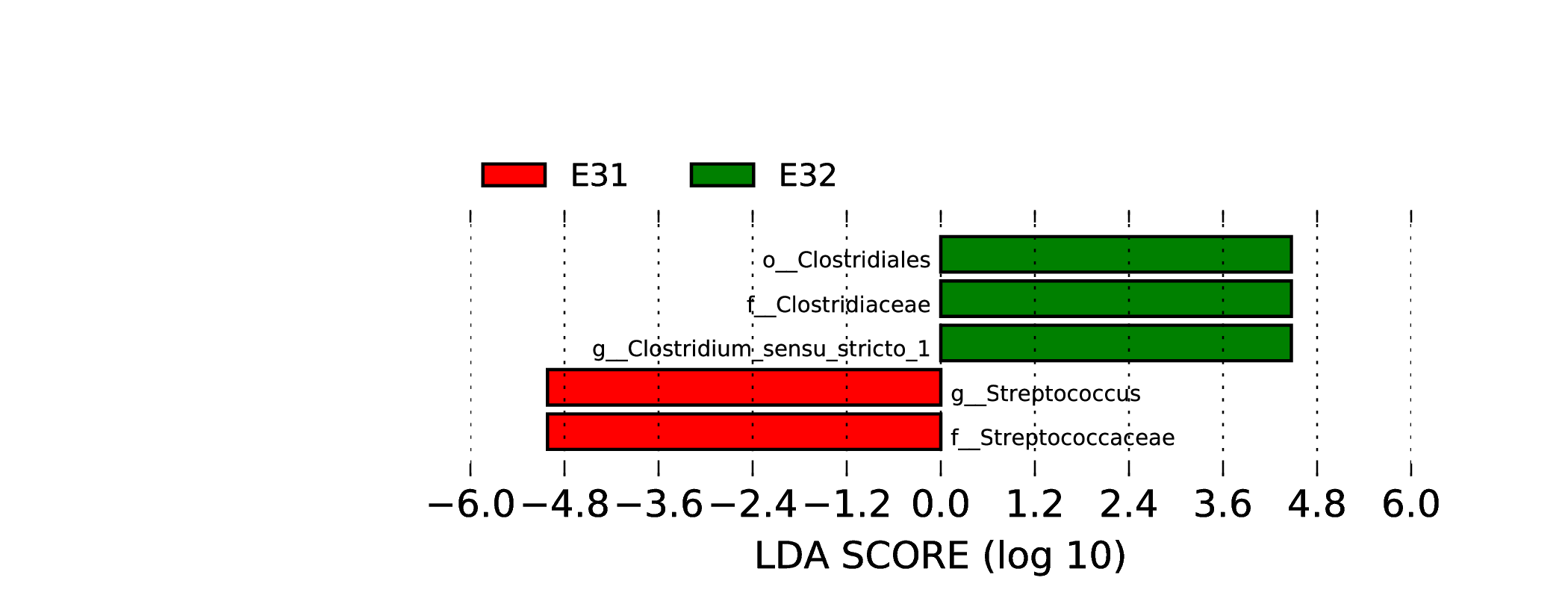

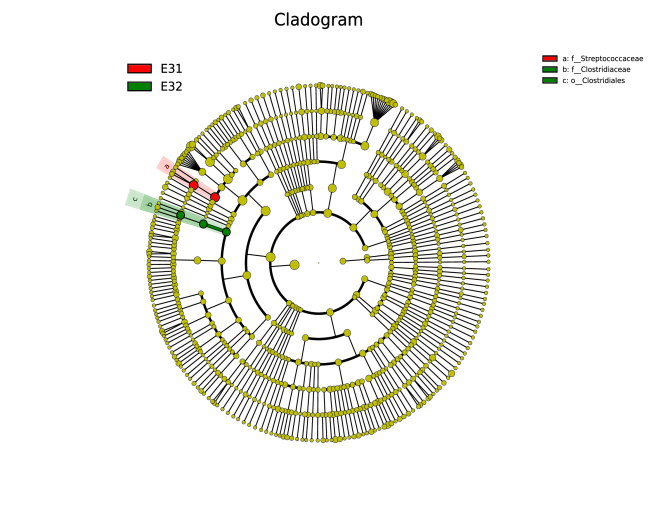


Figure j5 Figure j6

Figure j, j1.LDA score Histogram of differential microbiota of the two feeding patterns at 1d;j2.Cladogram of differential microbiota of the two feeding patterns at 1d;j3.LDA score Histogram of differential microbiota of the two feeding patterns at 3d;j4.Cladogram of differential microbiota of the two feeding patterns at 3d;j5.LDA score Histogram of differential microbiota of the two feeding patterns at 5d;j6.Cladogram of differential microbiota of the two feeding patterns at 5d.
